# Activation of the intrinsic fibroinflammatory program in adult pancreatic acinar cells triggered by Hippo signaling disruption

**DOI:** 10.1371/journal.pbio.3000418

**Published:** 2019-09-12

**Authors:** Jun Liu, Ming Gao, Michael Nipper, Janice Deng, Francis E. Sharkey, Randy L. Johnson, Howard C. Crawford, Yidong Chen, Pei Wang

**Affiliations:** 1 Department of Cell Systems & Anatomy, UT Health San Antonio, San Antonio, Texas, United States of America; 2 Department of Pathology, UT Health San Antonio, San Antonio, Texas, United States of America; 3 Department of Cancer Biology, Division of Basic Science Research, the University of Texas MD Anderson Cancer Center, Houston, Texas, United States of America; 4 Department of Molecular and Integrative Physiology & Internal Medicine, University of Michigan Health System, Ann Arbor, Michigan, United States of America; 5 Department of Epidemiology Biostatistics, UT Health San Antonio, San Antonio, Texas, United States of America; The Francis Crick Institute, UNITED KINGDOM

## Abstract

Damaged acinar cells play a passive role in activating pancreatic stellate cells (PSCs) via recruitment of immune cells that subsequently activate PSCs. However, whether acinar cells directly contribute to PSC activation is unknown. Here, we report that the Hippo pathway, a well-known regulator of proliferation, is essential for suppression of expression of inflammation and fibrosis-associated genes in adult pancreatic acinar cells. Hippo inactivation in acinar cells induced yes-associated protein 1 (YAP1)/transcriptional coactivator with PDZ binding motif (TAZ)-dependent, irreversible fibrosis and inflammation, which was initiated by Hippo-mediated acinar-stromal communications and ameliorated by blocking YAP1/TAZ target connective tissue growth factor (CTGF). Hippo disruption promotes acinar cells to secrete fibroinflammatory factors and induce stromal activation, which precedes acinar proliferation and metaplasia. We found that Hippo disruption did not induce cell-autonomous proliferation but primed acinar cells to exogenous pro-proliferative stimuli, implying a well-orchestrated scenario in which Hippo signaling acts as an intrinsic link to coordinate fibroinflammatory response and proliferation for maintenance of the tissue integrity. Our findings suggest that the fibroinflammatory program in pancreatic acinar cells is suppressed under normal physiological conditions. While transient activation of inflammatory gene expression during tissue injury may contribute to the control of damage and tissue repair, its persistent activation may result in tissue fibrosis and failure of regeneration.

## Abstract 2

The mechanisms by which epithelial cells orchestrate the intrinsic fibro-inflammatory response and cell proliferation during the repair of injured tissues remains unclear. A study of molecular and cellular changes in pancreatic acinar cells suggests that the Hippo pathway acts as an intrinsic link to coordinate fibro-inflammatory response and proliferation process in epithelial cells.

## Introduction

Tissue damage and repair is a complicated process involving interactions among epithelial, stromal, and immune cells, enabling well-coordinated inflammatory response to control damage and proliferation of epithelial cells to regenerate tissues. It is well established that the tightly controlled fibroinflammatory program in epithelial cells is transiently activated during organ damage to assist repair of injured tissues. However, the mechanisms by which epithelial cells orchestrate the intrinsic fibroinflammatory response and cell proliferation largely remain unclear. Deregulation of these processes contributes to persistent tissue damage and the development of fibrotic diseases.

As digestive enzyme production factories, when acinar cells are damaged, they may release enzymes that can cause severe detrimental effects in the abdominal cavity. It is plausible to speculate that acinar cells should have an intrinsic mechanism to quickly sense these changes and modulate their microenvironment to contain the damage and promote regeneration. Increasing numbers of studies have begun to investigate the active roles of acinar cells in regulating the inflammatory response [1,2,3]. Considering the fact that the Hippo pathway is regulated by soluble factors, cell-cell contact, and extracellular matrix (ECM) stiffness [4], it is tempting to speculate that acinar cells may utilize this pathway to sense these exogenous stimuli and actively translate them into signals to orchestrate the surrounding microenvironment.

The central kinases of the Hippo signaling pathway in mammals, large tumor suppressor 1 and 2 (LATS1/2), play important roles in inhibiting proliferation, promoting apoptosis, and inhibiting F-actin polymerization [5]. LATS1/2 directly phosphorylate downstream effectors yes-associated protein 1 (YAP1) or transcriptional coactivator with PDZ binding motif (TAZ) and restrict their activity via both cytoplasmic retention and ubiquitin-proteasome-mediated degradation. Once LATS1/2 are inactivated, unphosphorylated YAP1/TAZ translocate to the nucleus to initiate the transcription of many genes, including pro-proliferative and anti-apoptotic genes [6]. The Hippo pathway is frequently inactivated during acute tissue damage as well as in a broad range of fibrotic diseases, which has generally been believed to support tissue repair via promoting cell-autonomous proliferation through YAP1/TAZ. However, recent studies suggest that functions of the Hippo pathway are more diverse than expected. Enforced expression of constitutively activated mutants of YAP1/TAZ in cancer cells frequently induces expression of inflammatory cytokines, raising the possibility that the Hippo pathway might be a key player to link epithelial fibroinflammatory response and proliferation during tissue repair. However, whether the Hippo pathway is a physiological repressor for the intrinsic fibroinflammatory program in epithelia cells remains an unanswered question.

Recent clinical data support the existence of a continuum between recurrent acute pancreatitis (AP) and chronic pancreatitis (CP) [7]. CP is caused by a persistent inflammatory response that eventually leads to fibrosis and pancreatic failure. Currently, there are no specific treatments or prevention strategies for this disease [8]. Although premature intra-acinar trypsinogen activation has been considered a major pathogenic factor contributing to AP [9], the mechanisms driving pancreatic fibrosis—the characteristic feature of CP—during the progression from AP to CP remain unclear[10]. The fact that the nuclear expression of YAP1 and TAZ has been found in human pancreatic cancer and pancreatitis samples [11,12] strongly suggests that the Hippo pathway may play important roles in the pathogenesis of pancreatic disease.

Extensive evidence suggests that stromal cell activation state plays a central role in determining the outcome of tissue repair [13]. Although a number of studies have shown a passive role of damaged acinar cells in activating pancreatic stellate cells (PSCs) via recruitment of immune cells, the possibility that acinar cells may actively modulate the microenvironment to support sustained PSC activation has not been well studied [14,1]. Indeed, several key PSC activators such as transforming growth factor, beta (TGFβ) and connective tissue growth factor (CTGF) are up-regulated in acinar cells adjacent to the areas of fibrosis in pancreata of CP patients [15,16]. Therefore, it is important to understand the mechanisms mediating acinar-cell–PSC communication and to investigate their contribution to the pathogenesis of CP.

In this study, we used the Ptf1a^Cre-ER^/LoxP system in mouse pancreas and found that mice with acinar-specific deletion of Lats1/2 developed strong inflammation and surprisingly rapid and severe fibrosis in a YAP1/TAZ-dependent manner. We demonstrated that Hippo disruption induces expression of inflammation and fibrosis-associated genes in acinar cells, which directly contributes to stromal activation. In contrast to previous studies that focused on YAP1/TAZ-mediated cell proliferation and differentiation, we found that acinar fibroinflammatory activation occurs before YAP1/TAZ-driven cell proliferation. Our results grant new perspective of the physiological functions of the Hippo pathway and add an additional layer of complexity to the current model for the roles of Hippo signaling in tissue regeneration. We further identified a number of up-regulated pro-inflammatory and profibrotic genes in Lats1/2-deficient acinar cells, suggesting that these identified genes might be targets of YAP1/TAZ. Our data suggest that the Hippo pathway, in addition to its well-known function to control cell proliferation, plays an essential role in the inhibition of fibroinflammatory response in epithelial cells.

## Results

### The intact epithelial Hippo pathway is essential to suppress fibroinflammatory response in the adult pancreas

To investigate the roles of LATS1/2 in the pancreas, we genetically ablated *Lats1* and *2* genes specifically in pancreatic acinar cells at the adult stage. Through multiple steps of breeding (S1A Fig), we generated *Lats1* and *2* double knockout mice (PL: *Ptf1a*^*CreER*^*Lats1*^*fl/fl*^*Lats2*^*fl/fl*^*Rosa26*^*LSL-YFP*^), *Lats1* and *Lats2* single knockout mice (PL1KO: *Ptf1a*^*CreER*^*Lats1*^*fl/fl*^*Lats2*^*fl/+*^*Rosa26*^*LSL-YFP*^; PL2KO: *Ptf1a*^*CreER*^*Lats1*^*fl/+*^*Lats2*^*fl/fl*^*Rosa26*^*LSL-YFP*^), and flox-alleles–alone mice (Lats1/2: *Ptf1a*^*+/+*^*Lats1*^*fl/fl*^*Lats2*^*fl/fl*^*Rosa26*^*LSL-YFP*^). We used Cre alone (P: *Ptf1a*^*CreER*^*Rosa26*^*LSL-YFP*^) mice as controls. We administered 180 mg/kg/d of tamoxifen (TAM) to 6- to 12-week-old mice via intraperitoneal (i.p.) injection for 5 consecutive days. We found that PL mice became lethargic and started losing weight by 7 days after the last TAM injection (S1B Fig). At day 10, PL mice had a weight loss of 19.2%. We found a significant reduction of blood glucose levels in PL mice, suggesting a starvation phenotype (S1C Fig). We observed a continuous loss of acinar tissue and replacement by fibrous tissue over 14 days (S1D Fig).

**Fig 1:**
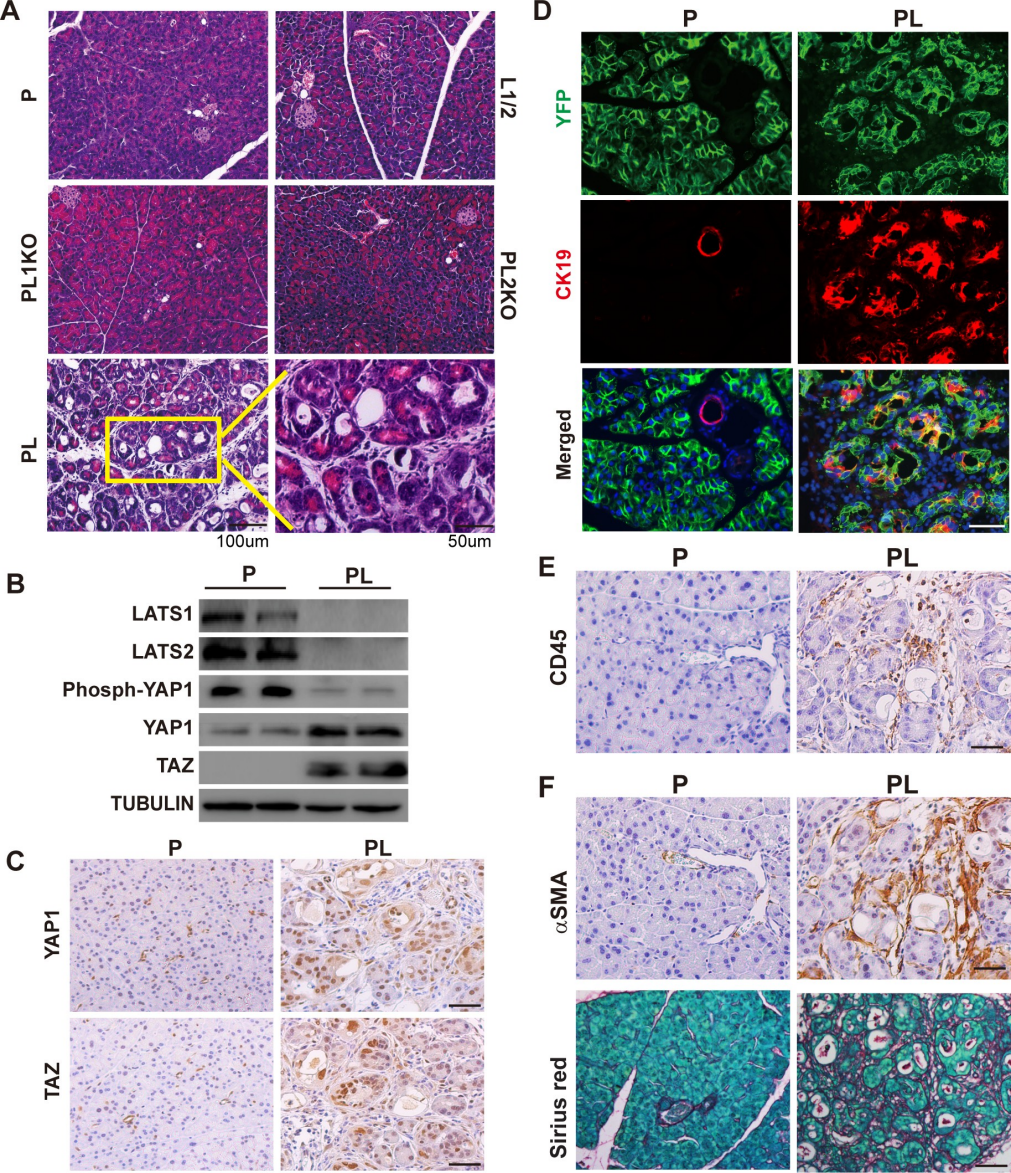
Acinar cell-specific *Lats1/2* deletions caused pancreatic inflammation and fibrosis in adult mice. P, PL, L1/2, PL1KO, and PL2KO mice were administrated with 180 mg/kg/d of TAM for 5 consecutive days via i.p., respectively (*n* = 6). Pancreata were collected at Day 5 after the final injection. (A) Histological morphology of P, L1/2, PL, PL1KO, and PL2KO mice. L1/2, PL1KO, and PL2KO mice were littermate controls. (B) Western blot showing the levels of LATS1, LATS2, phospho-YAP1, YAP1, and TAZ in P and PL mice. Tubulin was used as the internal control. (C) YAP1 and TAZ IHC staining in P and PL mice. (D) Immunofluorescence of YFP (Green) and CK19 (Red) in P and PL mice. Nuclei stained with DAPI (Blue). (E) P and PL pancreata stained with anti-CD45 antibody. (F) P and PL pancreata stained with anti-αSMA antibody, and Sirius Red, respectively. αSMA, α-smooth muscle actin; CD45, ; CK19, cytokeratin 19; IHC, immunohistochemistry; i.p., intraperitoneal; L1/2, ; LATS1, large tumor suppressor 1; P, control; PL, double knockout; PL1KO, *Lats1* knockout; PL2KO, *Lats2* knockout; TAM, tamoxifen; TAZ, transcriptional coactivator with PDZ binding motif; YAP1, yes-associated protein 1.

Histologically, PL mice exhibited severe damage in the pancreas, including acinar cell atrophy, inflammatory cell infiltration, and acinar-to-ductal metaplasia (ADM) at Day 5 after the last injection of TAM (Fig 1A). No such defects were observed in the pancreata of control groups (Fig 1A). Successful *Lats1/2* deletions in the pancreas were determined by PCR analysis (S2A Fig) and western blot (Fig 1B). The protein levels of downstream effectors YAP1/TAZ were increased dramatically in PL mice, while phospho-YAP1 levels were lower in PL mice (Fig 1B). YAP1 and TAZ nuclear translocations were detected in PL mice by immunohistochemistry (IHC) staining (Fig 1C, S2B Fig), suggesting that loss of LATS1/2 in pancreatic acinar cells induced YAP1/TAZ activation, similarly to other tissues [17]. Compared to the control P mice, we detected an increased expression of ductal cell marker cytokeratin 19 (CK19) in PL mice (Fig 1D). Yellow fluorescent protein (YFP^+^)/CK19^+^ cells were only observed in PL mice, suggesting that acinar cells underwent ADM, a common hallmark of pancreatitis (Fig 1D, S3A Fig). The antigen identified by monoclonal antibody Ki 67 (Ki67) expression was found in YFP^+^/CK19^+^ cells, suggesting that proliferation was activated during ADM (S3B Fig). We found apoptotic cells in PL mice by staining of cleaved caspase-3 (S3C Fig). Consistent with hematoxylin–eosin (HE) staining, we identified infiltration of immune cells in PL mice by cluster of differentiation antigen 45 (CD45) staining (Fig 1E, S3A Fig). We detected strong staining of α-smooth muscle actin (αSMA) and significant deposition of collagen in PL pancreata (Fig 1F, S3A Fig), suggesting the occurrence of PSC activation and pancreatic fibrosis. Taken together, these results strongly suggest that the loss of LATS1/2 in pancreatic acinar cells rapidly leads to pancreatic fibrosis, a major clinical feature of CP.

### *Hippo* disruption-induced pancreatic inflammation and fibrosis are YAP1/TAZ dependent

**Fig 2:**
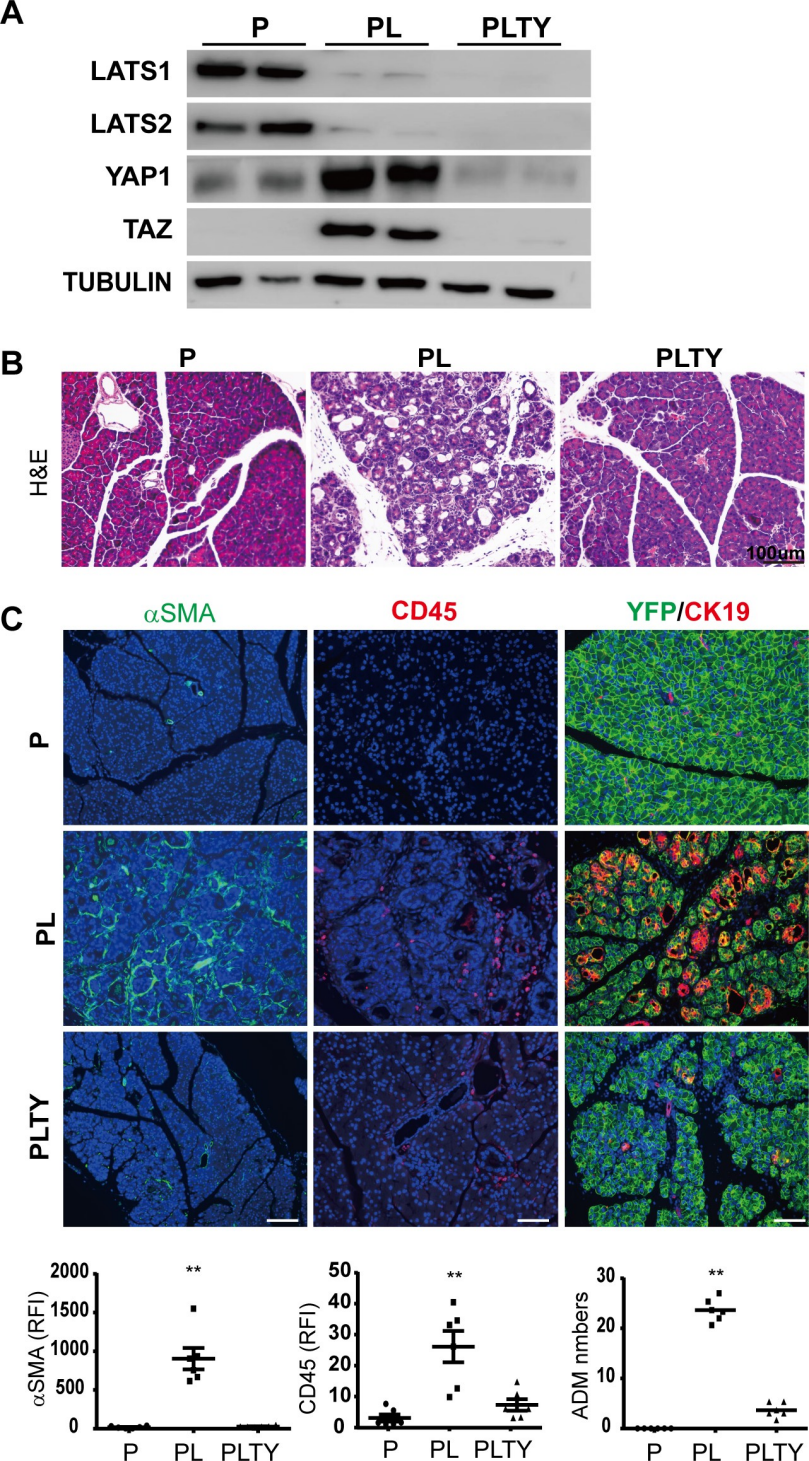
Loss of YAP1/TAZ in acinar cells rescues *Lats1/2*-deletion–induced pancreatic inflammation and fibrosis. P, PL, and PLTY mice were subjected to 180 mg/kg/d of TAM for 5 consecutive days via i.p. (*n* = 6). Pancreata were collected 5 days later. (A) Western blot of Lats1, Lats2, YAP1, and TAZ in P, PL, and PLTY mice. Tubulin was used as the internal control. (B) Histological examination by HE staining among P, PL, and PLTY groups. (C) Immunofluorescence staining of αSMA (Green), CD45 (Red), and ADM (Green + Red) among P, PL, and PLTY groups. Nuclei stained with DAPI (blue). Quantification of αSMA and CD45 immunostaining in P, PL, and PLTY mice showed 90.1% reduction of inflammation and 95.2% reduction of stromal reaction in PLTY mice compared with PL mice. ADM was quantified by counting YFP and CK19 double positive cell numbers. There was an 84.5% reduction of ADM in PLTY mice compared with PL mice (*n* = 6). **P < 0.01. Underlying numerical values can be found in S1 Data. αSMA, α-small muscle actin; ADM, acinar-to-ductal metaplasia; CD45, cluster of differentiation antigen 45; CK19, cytokeratin 19; HE, hemtoxylin–eosin; i.p., intraperitoneal; LATS1, large tumor suppressor 1; P, control; PL, double knockout; PLTY, quadruple deletions of Lats1/2 and YAP1/TAZ; TAM, tamoxifen; TAZ, transcriptional coactivator with PDZ binding motif; YAP1, yes-associated protein 1.

*Lats1/2* deletion in adult acinar cells dramatically up-regulated YAP1/TAZ in the pancreas and induced YAP1/TAZ nuclear translocation (Fig 1B and 1C, S2B Fig). Thus, we tested whether the inflammation and fibrosis in *Lats1/2* null pancreata were mediated by YAP1/TAZ. Loss of *YAP1/TAZ* in adult acinar cells (*Ptf1a*^*CreER*^*YAP1*^*fl/fl*^*TAZ*^*fl/fl*^, PTY) did not result in overt defect (S4A Fig). We then crossed PL mice with *Yap1*^*fl/fl*^*Taz*^*fl/fl*^ mice to generate *Ptf1a*^*CreER*^*Rosa26*^*LSL-YFP*^*Lats1*^*fl/fl*^*Lats2*^*fl/fl*^*Yap1*^*fl/fl*^*Taz*^*fl/fl*^ (PLTY) mice. The PLTY mice were administered with 180 mg/kg/d of TAM for 5 consecutive days to induce the quadruple deletions of *Lats1/2* and *YAP1/TAZ* genes in adult pancreatic acinar cells. P and PL mice were used as controls. All pancreata were harvested at Day 5 after the final injection of TAM (S4B Fig). The deletions of *Lats1*, *Lats2*, *Yap1*, and *Taz* were confirmed by western blot (Fig 2A, S4C Fig). HE staining revealed that ablation of *YAP1/TAZ* rescued the pancreatitis phenotype of PL mice (Fig 2B). PSC activation, immune cell infiltration, and ADM were remarkably reduced in PLTY mice when compared with PL mice (Fig 2C). These data strongly suggested that *Lats1/2*-deletion–induced inflammation and fibrosis depend on YAP1/TAZ.

### *Hippo* disruption-induced pancreatic inflammation is not initiated by acinar cell death

Inflammation in the pancreas is frequently induced by acinar cell injury [18]. To test whether inflammation in PL mice was induced by autonomous acinar cell death following Lats1/2 deletions, we decided to knock out *Lats1/2* in a small portion of acinar cells in the adult pancreas. If the inflammation was induced by acinar cell death due to Lats1/2 knockout, injured *Lats1/2* null acinar cells would be rapidly removed by immune cells and the pancreas should recover with the remaining wild-type acinar cells. We gave the mice single injections of different doses of TAM. *Ptf1a*^*CreER*^ induced chromosome recombination in a TAM dose-dependent manner (S5A Fig). PCR assays confirmed the occurrence of Cre-mediated *Lats1/2* deletions even 4 days after a single dose of 45 mg/kg TAM injection (S5A Fig). Strikingly, all mice developed inflammation 3 weeks after TAM injection, and the severity of the inflammation was correlated with TAM dose (S5C Fig). Acinar cells can recover in cerulein-induced AP mice; however, the pancreatic lesions developed in single-dose TAM-injected PL mice did not recover over time. This long-lasting phenotype suggested that acinar damage was not the major mechanism to initiate inflammation in PL mice.

**Fig 3:**
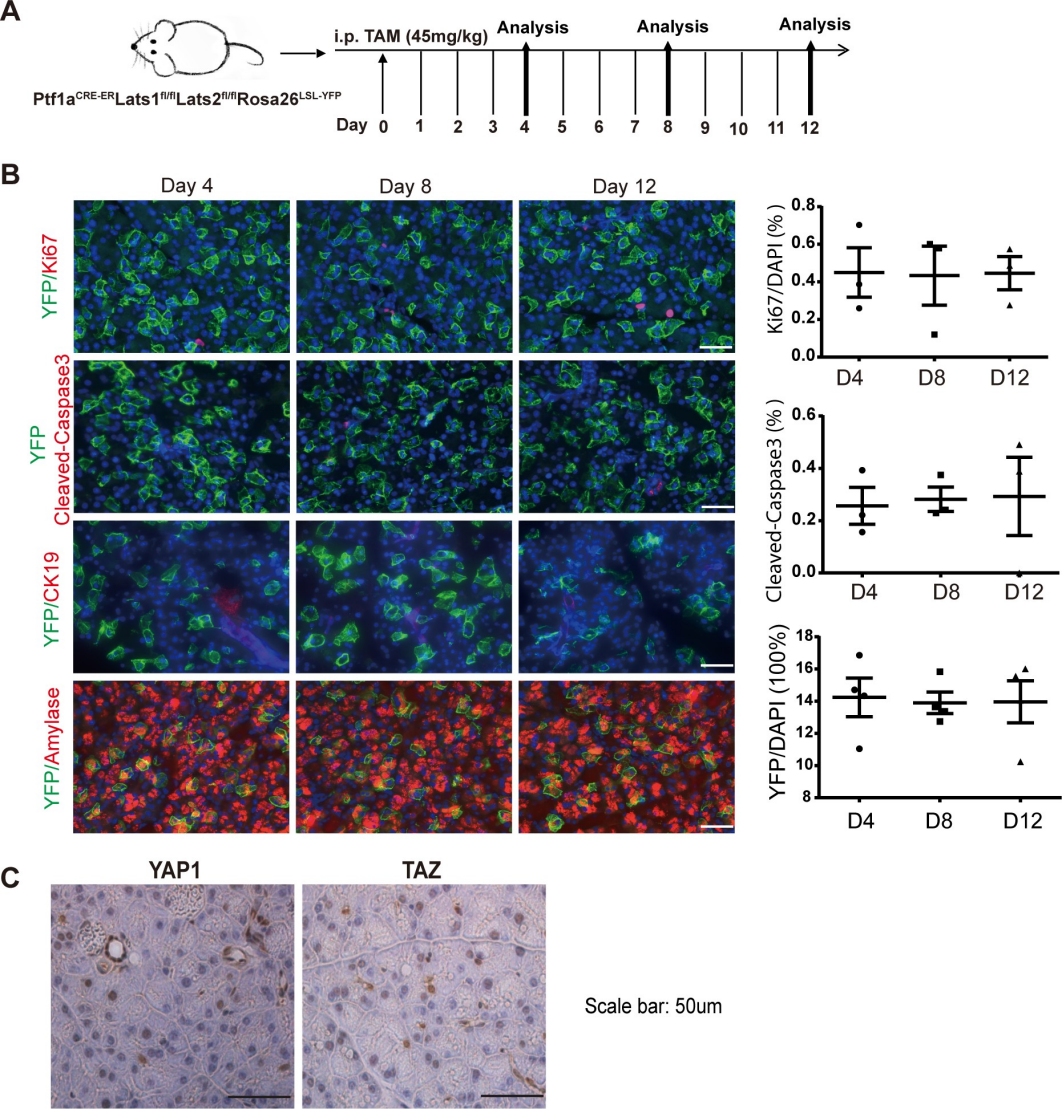
Loss of *Lats1/2* does not affect acinar cell proliferation, apoptosis, or ADM directly. (A) Schematic diagram of experimental design (*n* = 3). (B) PL mice were injected once with 45 mg/kg of TAM. Cell proliferation, apoptosis, and ADM were analyzed on Day 4, Day 8, and Day 12 by co-staining anti-YFP with anti-Ki67, anti-cleaved-caspase-3, anti-amylase, and anti-CK19 antibodies. Nuclei stained with DAPI (Blue). Percentages of Ki67, cleaved caspase-3 (*n* = 3), and YFP (*n* = 4) cells were quantified by IHC profiler score. Underlying numerical values can be found in S1 Data. (C) YAP1 and TAZ were detected by IHC in PL mice after injection with 45 mg/kg of TAM. ADM, acinar-to-ductal metaplasia; CK19, cytokeratin 19; IHC, immunohistochemistry; Ki67, antigen identified by monoclonal antibody Ki 67; LATS1, large tumor suppressor 1; P, control; PL, double knockout; TAM, tamoxifen; TAZ, transcriptional coactivator with PDZ binding motif; YAP1, yes-associated protein 1; YFP, yellow fluorescent protein.

We further chose mice that received a single low-dose (45 mg/kg) TAM injection for time course analysis because they showed Cre-mediated recombination in a fraction of individual acinar cells (S5B Fig), as determined by YFP expression. We co-stained YFP with cleaved-caspase-3 in the pancreata of PL mice at 4, 8, and 12 days after a single TAM injection (45 mg/kg) (Fig 3A) to test whether these cells underwent apoptosis. The results showed no YFP+ cells that were co-stained with cleaved-caspase-3 (Fig 3B). IHC assay revealed YAP1 and TAZ nuclear translocation in the PL pancreas, supporting the successful deletion of *Lat1&2* (Fig 3C). We further confirmed that *Lats1/2* deletions were restricted to YFP-positive cells by PCR assays after sorting by flow cytometry (S5D Fig). Collectively, these data indicate that loss of *Lats1/2* did not directly cause adult acinar cellular apoptosis.

### *Hippo* disruption does not directly induce autonomous proliferation in adult acinar cells

Because one of the best known functions of LATS1/2 is to control cellular proliferation and differentiation, we tested whether *Lats1/2* deletion induced ADM and proliferation of acinar cells. We did not observe YFP-positive cells expressing CK19 in the mice that received a single low-dose (45 mg/kg) TAM injection, indicating that no ADM occurred in *Lats1/2* null acinar cells (Fig 3B). We then examined Ki67 expression in these pancreata. No YFP^+^ cells expressed Ki67 at every time point. In addition, we quantified the number of YFP^+^ cells in these mice and found no significant differences during the 12-day period (Fig 3B). The different effects of low- and high-dose TAM suggested that Hippo disruption incudes ADM by non–cell-autonomous mechanisms. The smaller number of acinar cells with Hippo disruption induced by low-dose TAM require a longer time to create an ADM-prone microenvironment, while high-dose TAM induced Hippo disruption in a larger portion of acinar cells that can rapidly activate PSCs and immune cells to promote ADM.

To further confirm that Hippo disruption does not directly induce autonomous proliferation in adult acinar cells, we isolated acinar cells from PL mice that received 5 injections of TAM and cultured them in a collagen-based 3D model. *Lats1/2* deletions alone did not enhance the proliferation of acinar cells in 3D culture. However, in the presence of exogenous TGFα treatment, the most common method used to induce ADM in mouse acinar cells [19], the sizes of spheres formed by acinar cells with *Lats1/2* deletions were dramatically larger than those formed by the cells from control P mice (S5E Fig), suggesting that acinar cells with *Lats1/2* deletions did not induce autonomous cell proliferation but primed acinar cells to exogenous pro-proliferative stimuli from microenvironment to initiate cell proliferation.

### *Hippo* disruption in pancreatic acinar cells stimulates PSC activation

**Fig 4:**
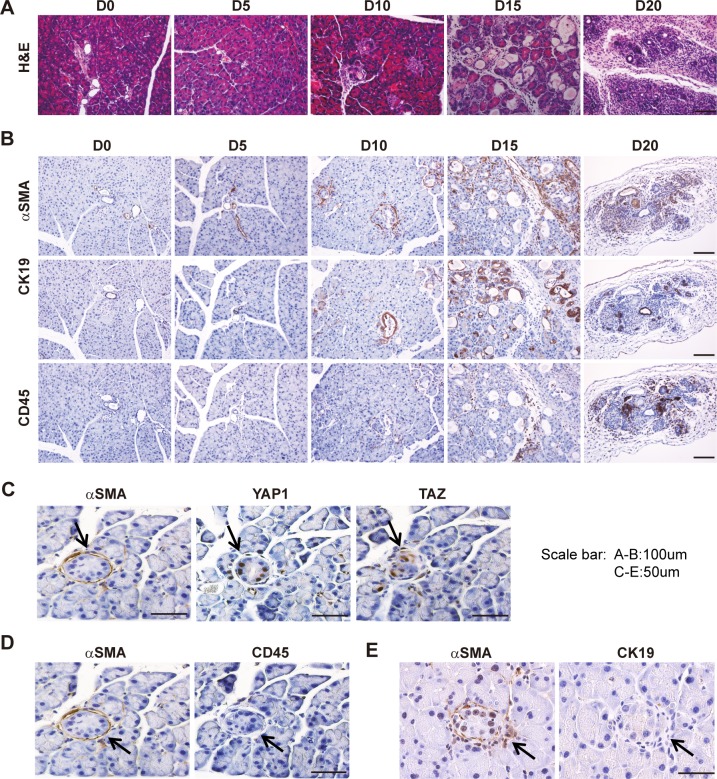
*Lats1/2* deletion in acinar cells results in PSC activation. PL mice were injected once with 180 mg/kg of TAM; pancreata were collected and embedded at Day 0, Day 5, Day 10, Day 15, and Day 20 after injection, respectively (*n* = 4–6). (A) Morphological changes over time (Day 0, Day 5, Day 10, Day 15, Day 20) were examined by HE staining. (B) Anti- αSMA, anti-CD45, and anti-CK19 antibodies were used to stain activated PSCs, immune cells, and ductal-like cells, respectively. (C) αSMA, YAP1, and TAZ IHC staining in consecutive sections at Day 10. (D) αSMA and CD45 IHC staining in consecutive sections at Day 10. (E) αSMA and CK19 IHC staining in consecutive sections at Day 10. αSMA, α-smooth muscle actin; CD45, cluster of differentiation antigen 45; CK19, cytokeratin 19; HE, hematoxylin–eosin; IHC, immunohistochemistry; LATS1, large tumor suppressor 1; PL, double knockout; PSC, pancreatic stellate cell; TAM, tamoxifen; TAZ, transcriptional coactivator with PDZ binding motif; YAP1, yes-associated protein 1.

To achieve a longer and clearer time window to analyze the temporal sequences of alterations in PL mice, we gave the mice a single TAM injection (180 mg/kg) and analyzed the pancreata at Days 0, 5, 10, 15, and 20. The pancreata of PL mice almost disappeared by Day 20. At Day 15, the phenotype was similar to what we observed at Day 5 for mice with 5 injections of TAM (Fig 4A). CK19, αSMA, and CD45 staining by IHC in consecutive sections of PL mice was positive in all lesions at Day 15, indicating the presence of ADM, PSC activation, and immune cell infiltration (Fig 4B, S6A Fig). However, the small dispersed lesions at Day 10 in PL pancreata with YAP1/TAZ nuclear accumulation were surrounded by αSMA-positive stromal cells, implying that PSCs might be activated by the direct communication with *Lats1/2* null acinar cells (Fig 4C). Indeed, many of these lesions were not accompanied by CD45-positive cells (Fig 4D). This was further confirmed by co-staining αSMA with CD45 (S6B Fig), highlighting the fact that PSCs were activated before significant infiltration of immune cells. These lesions retained acinar morphology and were negative for CK19, suggesting that PSCs were activated in the absence of ADM (Fig 4E). Together, our data indicate that activation of PSCs was an early event in PL mice.

#### Macrophages are polarized toward an M2 phenotype in the *Lats1/2* null pancreas

**Fig 5:**
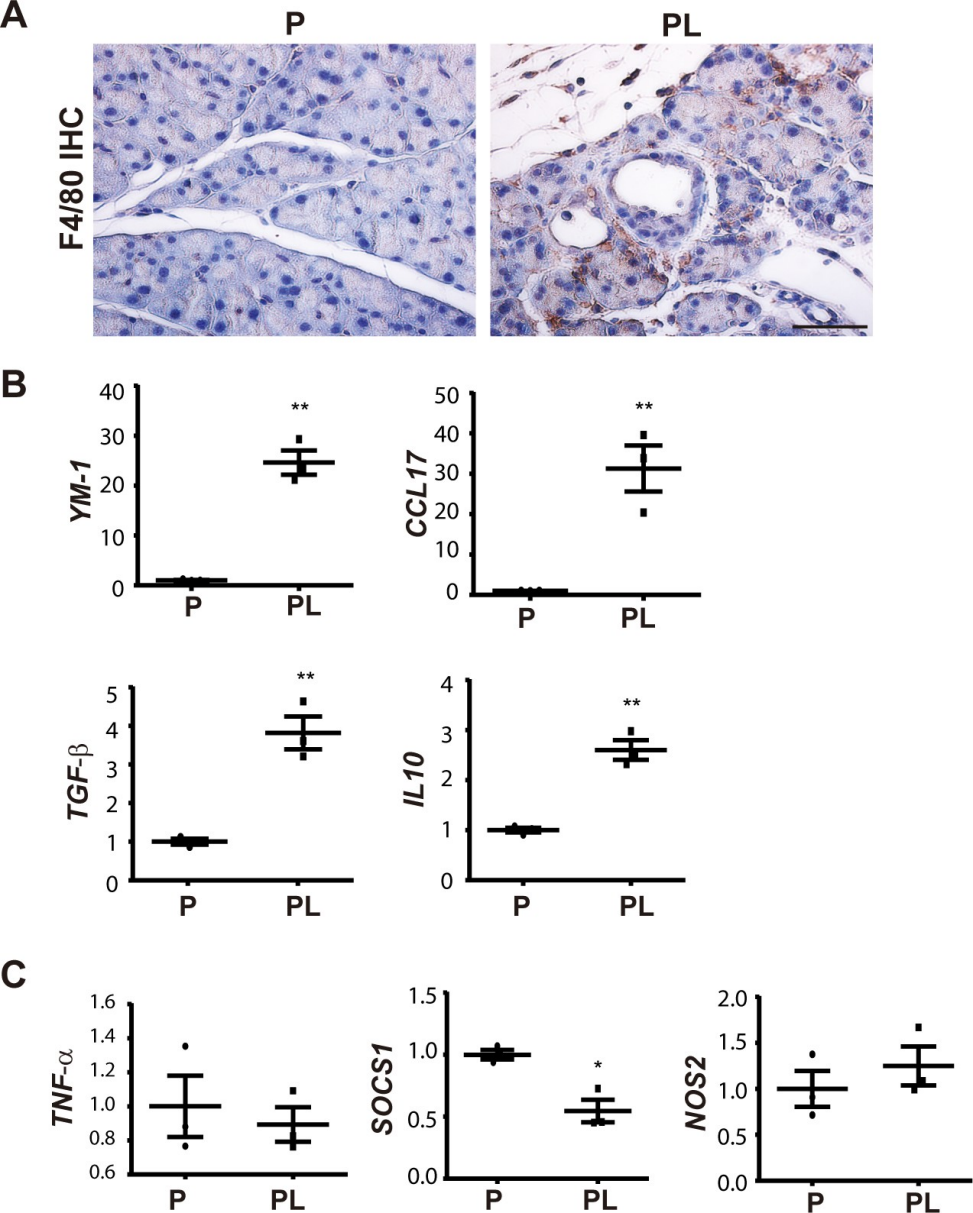
Loss of *Lats1/2* induces M2 macrophage polarization. P and PL mice were treated with 180 mg/kg/d of TAM for 5 consecutive days; cells were isolated from both groups 4 days later (*n* = 3). (A) F4/80 IHC staining in P and PL mice on Day 4 after TAM injection. (B) M2 macrophage-specific genes *Ym-1*, *Ccl17*, *Tgf-β* and *IL10* were elevated in sorted macrophages of PL mice. (C) M1 macrophage-specific genes *Tnf-α*, *Socs1*, and *Nos2* were not significantly changed. **P < 0.01, *P < 0.05. Underlying numerical values can be found in S1 Data. IHC, immunohistochemistry; LATS1, large tumor suppressor 1; P, control; PL, double knockout; TAM, tamoxifen.

Immune cell infiltration increased dramatically in Day 4 PL pancreata after 5 doses of TAM injection (S7A Fig). Unlike the tissue damage-induced M1 polarization of macrophages in AP, alternatively activated macrophages (M2) are dominant in CP, and activated PSCs are important inducers of M2 polarization [20]. Thus, we examined which macrophages were present in PL mouse. F4/80 staining revealed an increased number of macrophages in the pancreata of PL mice (Fig 5A). We used flow cytometry to sort CD45+CD11b+F4/80+ macrophages from the pancreata of P and PL mice (S7B Fig) 4 days after final TAM injection to examine the expression of a panel of M1- and M2-accociated genes [21]. Compared with the control P mice, macrophages from PL mice displayed a dramatic up-regulation of M2 macrophage-associated gene *Ym1* (Fig 5B). Other M2 macrophage-associated cytokines and chemokines, including *Il10*, *Tgf-β1*, and *Ccl17*, also showed significant up-regulation (Fig 5B). M1 macrophage-associated genes such as *Tnf-α*, *Sosc1*, and *Nos2* showed no significant expression change (Fig 5C), and *Il-1β* was barely detectable by quantitative PCR (qPCR). These data revealed that macrophages were rapidly polarized toward an M2 phenotype upon YAP1/TAZ activation in adult acinar cells.

### *Hippo* disruption activates the fibroinflammatory program in acinar cells

The observation that activation of PSCs was an early event in PL mice provoked us to examine whether Hippo pathway disruption induced the activation of fibrosis and inflammation-associated genes in acinar cells to modulate the microenvironment. Thus, we performed RNA sequencing (RNA-seq) analysis of pancreata from control P and mutant PL mice. To increase the number of *Lats1/2* null cells, control P mice and PL mice were subjected to180 mg/kg/d of TAM for 5 consecutive days. Tails of pancreata from P mice were harvested at Day 1 and used as the control. Tails of pancreata from PL mice were harvested at Days 1, 2, and 3 (*n* = 3–5 for each time point) for RNA extraction and RNA-seq library preparation. Histological examination showed no visible defects in Day 1, 2, or 3 PL pancreata (S8A Fig). There was no positive staining of CD45, αSMA, or CK19 at Day 2, but there was some at Day 3 (S8B Fig).

**Fig 6:**
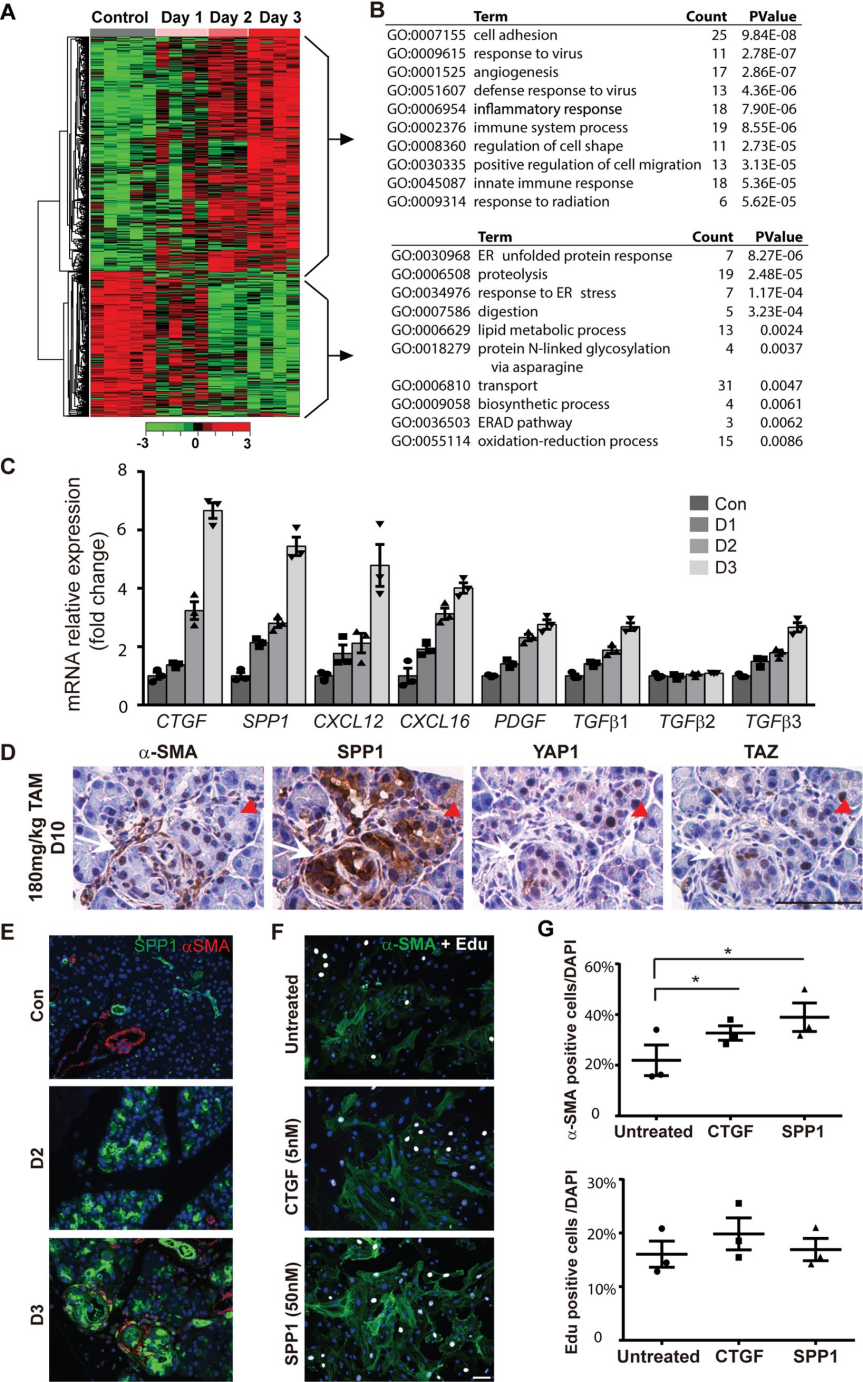
RNA-seq analysis of LATS1/2 regulated genes in mouse pancreas. (A) Heat map analysis showed differential gene expression patterns at different days (*n* = 3–5). (B) Left: K-means clustering analysis showed the gene expression patterns of 2 classes (up-regulated and down-regulated genes) that were changed with time. Right: GO term analysis of the function of top up-regulated and down-regulated genes. (C) Validation of profibrotic gene expressions by qPCR. (D) The small lesions were stained with anti- αSMA, anti-SPP1, anti-YAP1, and anti-TAZ antibodies in consecutive sections. White arrow indicates region in which αSMA is already present, while red arrow indicates region in which SPP1 expression has occurred but αSMA is not yet present. (E) Co-stain of αSMA (Red) with SPP1 (Green) in P and PL (Day 2 and Day 3) mice by immunofluorescence. (F) Isolated PSCs were treated with CTGF (5 nM) or SPP1 (50 nM) for 3 days (*n* = 3). Untreated group serves as the control. Anti-αSMA antibody was used to stain activated PSCs (Green). Cell proliferation was evaluated by EdU incorporation (White). Nuclei stained with DAPI (Blue). (G) SPP1 IHC staining in control, AP, and CP mice. Quantitative analysis for αSMA and EdU incorporation in CTGF or SPP1-stimulated cells (*n* = 3). *P < 0.05. Underlying numerical values can be found in S1 Data. αSMA, α-smooth muscle actin; AP, acute pancreatitis; CP, chronic pancreatitis; CTGF, connective tissue growth factor; EdU, 5-ethynyl-2'-deoxyuridine; IHC, immunohistochemistry; LATS1, large tumor suppressor 1; P, control; PL, double knockout; PSC, pancreatic stellate cell; qPCR, quantitative PCR; RNA-seq, RNA sequencing; SPP1, secreted phosphoprotein 1; TAZ, transcriptional coactivator with PDZ binding motif; YAP1, yes-associated protein 1.

We compared transcriptomes from RNA-seq data from PL mice at Days 1, 2, and 3 after TAM injection to control P mice and obtained 40, 405, and 765 differentially expressed genes (DEGs), respectively, using the significance criterion (fold-change > 2; adjusted *P* < 0.05; and read per kilobase of transcript per million reads mapped [RPKM] > 2). A large number of genes with expression changes were found in Day 2 PL pancreata (Fig 6A), although no significant PSC activation or immune cell infiltration was observed at this time point. We performed K-means clustering analysis and identified 2 representative clusters: 300 up-regulated genes and 232 down-regulated genes (Fig 6B). Several known YAP1/TAZ targets, including *Ctgf*, *Cyr61*, and *Spp1*, were among the up-regulated genes (S8C Fig). Gene ontology (GO) term analysis with the 300 up-regulated genes showed that 5 out of the top 10 GO terms were related to immune response, which is consistent with the phenotype of PL mice. On the other hand, down-regulated genes were related to endoplasmic reticulum and digestion, which are associated with normal acinar cell function (Fig 6B).

We specifically analyzed GO terms regarding growth factor activity (GO:0008083), cytokine activity (GO:0005125), and chemokine activity (GO:0008009), and identified a total of 12 up-regulated genes (*Bmp1*, *Ctgf*, *Cxcl12*, *Mia*, *Pdgfα*, *Tgfβ1*, *Tgfβ3*, *Timp1*, *Ccl12*, *Cxcl10*, *Cxcl14*, and *Spp1*). Interestingly, several genes involved in PSC activation (*Tgfβ* and *Pdgf*α) and fibrogenesis (*Spp1*, *Ctgf*, *Cxcl12*, and *Cxcl16*) were significantly up-regulated at Day 2, before immune cell infiltration, suggesting a potential function of acinar cell YAP1/TAZ in direct regulation of PSC activation and pancreatic fibrosis. We validated the expression changes of *Ctgf*, *Spp1*, *Cxcl12*, *Cxcl16*, *Pdgf*α, *TGFβ1*, *TGFβ2*, and *TGFβ3* by qPCR (Fig 6C). Except for *TGFβ2*, all of these genes showed gradual up-regulation during the time course, strongly supporting that they may be direct targets of YAP1/TAZ.

Among these genes, the up-regulation of secreted phosphoprotein 1 (*Spp1*) was the most dramatic. Even at Day 1 after TAM injection, we detected an about 2-fold increase of *Spp1* compared to the control P mice. *Spp1* has been suggested as a potential target of YAP1/TAZ [22] and is also strongly associated with TGF*β* function and hepatic stellate cell (HSC) activation [23]. Thus, we performed immunostaining of SPP1 on consecutive sections of Day 10 PL pancreata with one dose of TAM (180 mg/kg). We found that SPP1 staining correlated well with αSMA, YAP1, and TAZ staining in small lesions (Fig 6D, S9B Fig). SPP1 was strongly positive, while no apparent αSMA staining surrounded acinar cells at Day 2 and Day 3 after 5 doses of TAM (Fig 6E). Immunofluroescent staining and reverse transcription (RT)-PCR for SPP1 in P, PL, and PLYT mice pancreata after TAM treatment showed that SPP1 up-regulation due to Hippo pathway inactivation was dependent on YAP1/TAZ (S9C and S9D Fig). These findings suggest that up-regulation of SPP1 by YAP1/TAZ in pancreatic acinar cells may precede PSC activation. To further test whether SPP1 can activate PSCs, we isolated the primary PSCs by density gradient centrifugation to directly test the effect of SPP1 on PSC activation [24]. The purity of the PSCs was confirmed by Glial fibrillary acidic protein (GFAP) staining (S9A Fig). The primary PSCs were treated with SSP1 recombinant proteins (50 nM) for 3 days. CTGF is a well-known YAP1/TAZ target that can activate PSCs and is up-regulated in PL mice. We used CTGF recombinant protein as the positive control. SPP1 treatment significantly increased αSMA-positive cell numbers, similarly to what we observed in the CTGF treated group, suggesting that SPP1 can trigger PSC activation (Fig 6F). SPP1 treatment did not significantly enhance the proliferation of PSCs as determined by EdU staining (Fig 6G). We noticed that αSMA-positive cells and EdU-positive cells did not always overlap (Fig 6F), suggesting that they might be two uncoupled events in PSC activation. Together, these data supported the notion that SPP1 might be an important soluble factor secreted by acinar cells with Lats1/2 deletions to activate PSCs.

### Targeting the fibroinflammatory program in acinar cells attenuates *Hippo* disruption-induced pancreatic inflammation and fibrosis

**Fig 7:**
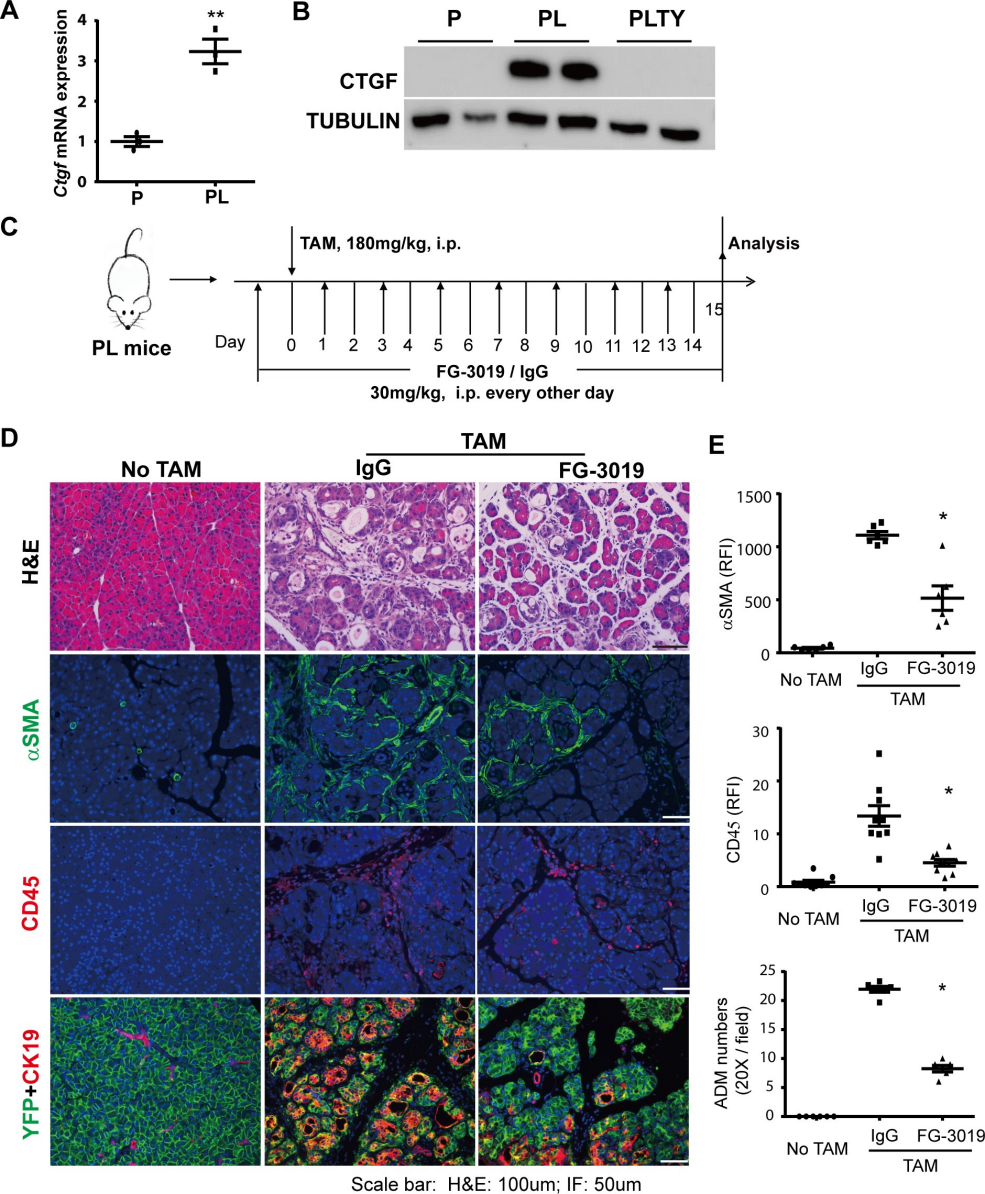
Depletion of CTGF partially abolishes pancreatic inflammation and fibrosis in *Lats1/2* double knockout mice. (A) *Ctgf* mRNA expression in P and PL mice (*n* = 3). **P < 0.01. (B) Western blot of CTGF protein levels in P, PL, and PLTY mice. Tubulin served as the internal control. (C) Schematic diagram of experimental design (*n* = 6 per group). Mice without TAM injection served as control group. FG-3019: CTGF-neutralizing antibody. (D) Top: histological examination by HE staining; center: immunofluorescent staining of αSMA (Green); bottom: immunofluorescent staining of CD45 (Red). Nuclei stained with DAPI (blue). (E) Quantitative analysis of FG-3019 treatment effects on Lats1/2 deletion-induced ADM, PSC activation, and immune cell infiltration in the mouse pancreas (*n* = 6); *P < 0.05. Underlying numerical values can be found in S1 Data. αSMA, α-smooth muscle actin; ADM, acinar-to-ductal metaplasia; CD45, ; CTGF, connective tissue growth factor; HE, hematoxylin–eosin; IgG, human Immunoglobulin G control; LATS1, large tumor suppressor 1; P, control; PL, double knockout; PLTY, quadruple deletions of Lats1/2 and YAP1/TAZ; PSC, pancreatic stellate cell; TAM, tamoxifen; TAZ, transcriptional coactivator with PDZ binding motif; YAP1, yes-associated protein 1.

To test whether the activation of the intrinsic fibroinflammatory program in acinar cells contributed to Hippo disruption-induced pancreatic inflammation and fibrosis, we performed a rescue experiment using CTGF-neutralizing antibody FG-3019 (FibroGen, Inc.) [25]. CTGF is the best known YAP1/TAZ target gene and has been documented to play a central role in PSC activation [26] and fibrosis [27]. We found that *Ctgf* mRNA expression was up-regulated at an early time point (Day 2) after *Lats1/2* deletions (Fig 7A). In addition, CTGF protein level was significantly decreased in PLTY mice compared to PL mice (Fig 7B, S10A Fig). We injected PL mice with FG-3019 or FG-hulgG (control, human Immunoglobulin G) for 2 days (i.p. 30 mg/kg/d), followed by a single TAM (180 mg/kg) injection. After this, the mice received FG-3019 or FG-hulgG injection every other day and were euthanized at Day 15 (Fig 7C). The HE staining revealed that CTGF depletion partially attenuated inflammation and fibrosis in PL mice (Fig 7D). FG-3019-treated mice achieved 61.7%, 53.5%, and 56.7% reduction of immune cell infiltration, stromal reaction, and ADM, respectively, compared with the FG-hulgG-treated group (Fig 7E), suggesting that neutralization of CTGF partially rescued the pancreatitis induced by *Lats1/2* deletions in adult acinar cells. To examine whether neutralizing CTGF affected the health of the mice, we treated another group of mice (*n* = 6) for 30 days. Half of the FG-3019-treated mice maintained their body weight, while all FG-hulgG-treated mice lost weight by Day 12 (S10B Fig).

### Pancreatitis mouse models exhibit *Hippo* disruption-induced molecular features

**Fig 8:**
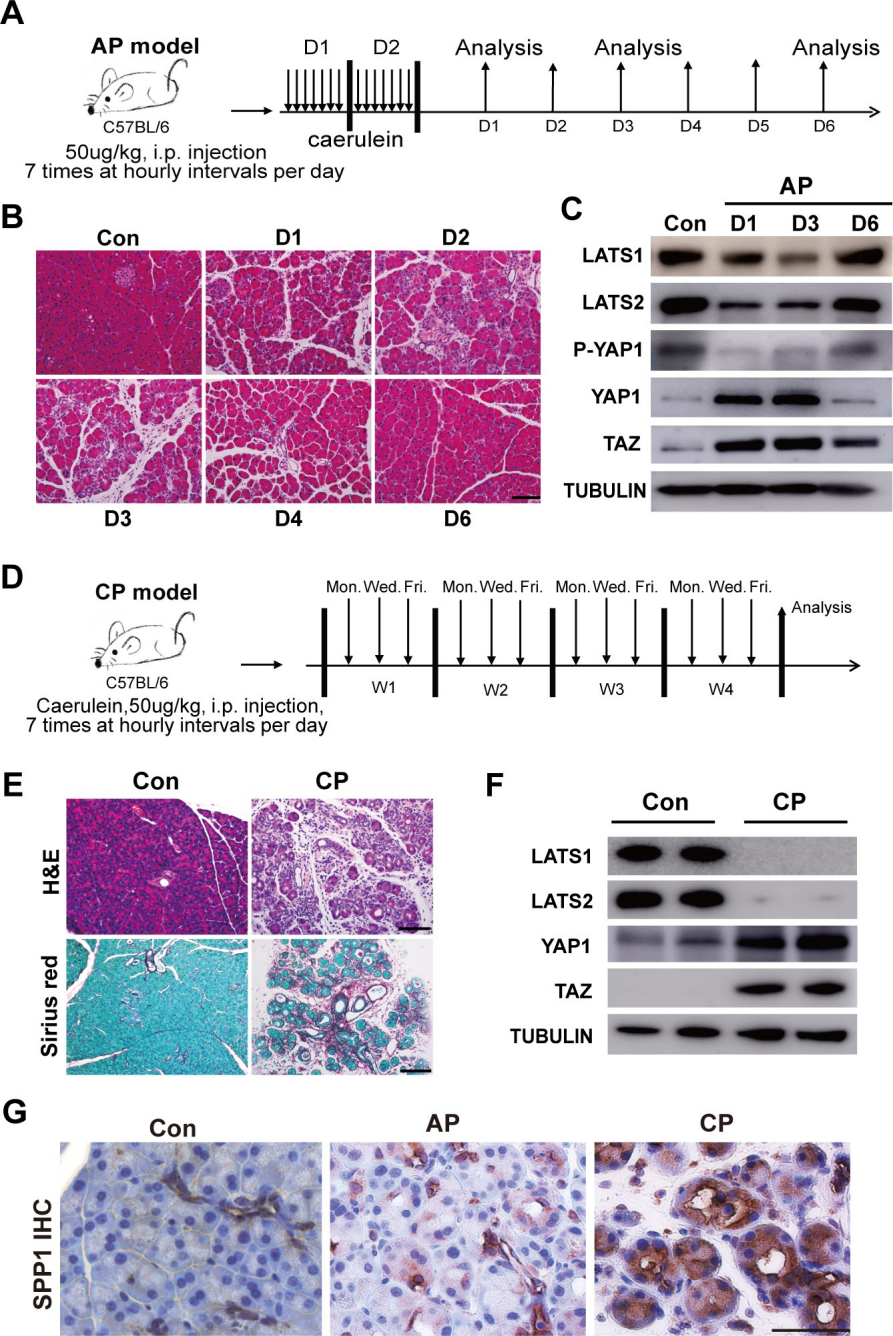
The Hippo signaling pathway is involved in inflammation and fibrosis in caerulein-induced pancreatitis mouse model. (A) Schematic diagram of induction of AP. *n =* 3. (B) The AP was analyzed by HE examination on Day 1, Day 2, Day 3, Day 4, and Day 6 after the induction. (C) Western blot of LATS1, LATS2, P-YAP1, YAP1, and TAZ in untreated and AP mice. Untreated mice served as the control group. Tubulin was used as the internal control. (D) Schematic diagram of induction of CP (*n =* 4). (E) HE examination of CP mice. Untreated mice served as the control group. Sirius Red was used to detect collagen deposition in CP mice. (F) Western blot of LATS1, LATS2, YAP1, and TAZ in untreated and CP mice. AP, acute pancreatitis; CP, chronic pancreatitis; HE, hematoxylin–eosin; LATS1, large tumor suppressor 1; P-YAP1, phospho-YAP1; TAZ, transcriptional coactivator with PDZ binding motif; YAP1, yes-associated protein 1.

To test potential involvement of the Hippo pathway in pancreatitis, we examined the histological alterations and the expression patterns of Hippo pathway core components in time course analysis of caerulein-induced AP (Fig 8A). Pancreatic inflammation was observed from Day 1 to 3, and recovery started at Day 4. By Day 6, the pancreas appeared normal (Fig 8B). Interestingly, western blot results revealed that the protein levels of Lats1/2 were significantly down-regulated from Day 1. By Day 6, the expression of both LATS1 and LATS2 recovered. In addition, the level of phospho-YAP1 (P-YAP1) showed a similar trend as LATS kinase. Inversely, YAP1/TAZ were increased from Day 1, and when the pancreas recovered, YAP1/TAZ went down back to normal levels (Fig 8C, S11A Fig). YAP nuclei staining was observed at Day 1 in the AP pancreas (S11B Fig). These data indicated a strong relationship between the Hippo signaling pathway and inflammatory response. CP was induced by repetitive episodes of AP for 4 weeks (Fig 8D), resulting in pancreatic fibrosis and increased collagen deposition, which was stained by Sirius Red (Fig 8E). Western blot data clearly showed significant reduction of LATS1/2 levels and elevated levels of YAP1/TAZ in the CP group compared to control mice (Fig 8F, S11C Fig), suggesting that Hippo signaling is also involved in pancreatic fibrosis. SPP1 is expressed in pancreatic ductal tissues and undifferentiated pancreatic precursors [28], as well as in pancreatic cancer [29]. However, its role in pancreatitis is unknown. We found SPP1-positive staining in ductal cells in control pancreata, but also in acinar cells in caerulein-induced AP and CP, with much stronger staining in CP pancreata (Fig 8G). Taken together, we found that the Hippo signaling pathway is dynamically regulated during caerulein-induced pancreatitis, indicating a potential function of Hippo signaling in modulating pancreatic inflammation and fibrosis.

## Discussion

The fibroinflammatory program and cellular proliferation in epithelial cells is critical for tissue repair. However, the key molecules linking these processes largely remain unclear. Cobo and colleagues [30] recently reported that nuclear receptor subfamily 5, group A, member 2 (NR5A2), a key transcriptional factor regulating acinar identity, controls an inflammatory program in acinar cells, establishing connection between the transcriptional networks of cellular differentiation and suppression of inflammatory programs. Our study revealed another important player that links epithelial fibroinflammatory signaling with cellular proliferation pathways for rapid response to tissue damage and subsequent tissue regeneration. We observed that acinar cells with disrupted Hippo signaling are surrounded by activated PSCs at early time points after acinar-specific Lats1/2 deletion. These data emphasize the critical role of the Hippo pathway in mediating epithelial–stromal interactions to modulate the microenvironment. Hippo disruption did not affect the expression of *Nr5a2* in acinar cells, suggesting the existence of multiple mechanisms in acinar cells to inhibit the intrinsic fibroinflammatory program under physiological conditions. Importantly, our data showed that Hippo disruption primed acinar cells to exogenous pro-proliferative stimuli without directly inducing cell-autonomous proliferation, highlighting a well-orchestrated scenario in which a Hippo disruption-induced fibroinflammation program signals surrounding stromal cells to support proliferation of epithelial cells.

Unlike other exocrine tissues, pancreatic acinar cells produce large amounts of digestive enzymes to aid digestion. Although these enzymes are packaged as zymogens which normally become active enzymes in the gut, damage to the pancreas can allow them to become active within the pancreas and have detrimental effects on the pancreas itself as well as other internal organs. A sensitive and well-coordinated system is required to contain the damage and promote regeneration in the pancreas. Our study showed that disruption of the Hippo pathway in acinar cells can quickly activate PSCs and recruit immune cells to the pancreas, suggesting that this pathway acts as a core regulator of the fibroinflammatory program in epithelial cells to restrict the spread of active enzymes by ECM deposition from PSCs and to control damage by recruiting immune cells.

RNA-seq data showed that deletion of *Lat1&2* induced early up-regulation of fibroinflammatory-reaction–associated genes, further supporting that Hippo signaling is a key repressor for the intrinsic fibroinflammatory program in acinar cells under physiological conditions. Consistent with previous studies that showed that SPP1 is a potential YAP1/TAZ target, we found that it is up-regulated in acinar cells soon after *Lats1/2* deletion. SPP1 is a known mediator of inflammation and fibrogenesis in different organs [31,32]. SPP1 treatment enhanced the activation of isolated primary PSCs. Moreover, in PL mice, SPP1 up-regulation co-localizes with YAP- and TAZ-activated acinar cells and strongly correlates with the activation of PSCs. These findings revealed a potential mechanism by which the Hippo pathway mediates epithelial–stromal communications via regulating the fibroinflammatory program in epithelial cells.

A recent study discovered that *Lats1/2* knockout cancer cells secreted DNA containing exosomes to promote T help cell type 1 response and created an antitumor inflammatory environment [33]. However, we found rapid accumulation of T help cell type 2 response-associated M2 macrophages in our mouse model, suggesting a different mechanism. Acinar cells have been thought to have a passive role in promoting inflammation via release of damage-associated molecular patterns when damaged [34]. In this sense, it is unexpected that deletion of *Lats1/2* in adult acinar cells would induce sterile inflammation because it is not intuitive to connect tissue damage to the knockout of *Lats1/2* based on their well-known functions [35]. Indeed, time course analysis revealed that PSC activation and immune cell infiltration happened well before acinar cell death after *Lats1/2* deletion. In addition, unlike the rapid tissue recovery from AP caused by acinar cell damage, deleting Lats1/2 in a small fraction of adult acinar cells by low-dose TAM administration induced persistent pancreatic inflammation, suggesting that these cells were not rapidly removed by immune cells. These data strongly suggest that pancreatic inflammation and fibrosis after acinar-specific *Lats1/2* deletion are not initiated by acinar cell damage. Nevertheless, whether the acinar cells with *Lats1/2* deletions are more susceptible to apoptosis under an inflammatory microenvironment should be further examined, because this might help to explain the severe phenotype in our PL model.

The observation that PSC activation occurs before immune cell infiltration suggests that disrupted Hippo signaling in acinar cells may directly contribute to PSC activation during tissue damage. While this process might contribute to epithelial regeneration for repair of injured tissues, its deregulation might lead to persistent PSC activation, resulting in excessive deposition of ECM in the gland. Indeed, our mouse model with deletion of Hippo core kinases LATS1/2 in adult pancreatic acinar cells induces prolonged activation of an epithelial fibroinflammatory program. These mice developed severe, YAP1/TAZ-dependent pancreatic fibrosis and inflammation at a strikingly fast speed that has not been seen in other mouse models [36,37,38]. A well-known YAP1/TAZ target, CTGF, was significantly up-regulated in PL mice. Depletion of CTGF with neutralizing antibody ameliorated the pancreatic fibrosis in PL mice. Corroborating with the clinical observation that CTGF expression is induced in acinar cells in human CP [15], these data suggest that targeting genes induced by Hippo disruption might be a promising strategy for CP treatment. Of note, the concept that YAP1/TAZ-mediated epithelia–stromal interaction plays an important role in tissue fibrosis may be applied to other tissues. TAZ induces the expression of Indian hedgehog (Ihh) to activate HSCs and promote nonalcoholic steatohepatitis-associated fibrosis and inflammation [39]. Nevertheless, Ihh does not activate the proliferation or ECM synthesis of PSCs [40], and Hippo disruption did not induce Ihh expression in adult acinar cells.

The ability of *Lats1/2* null acinar cells to directly activate PSCs provides an explanation for the rapid progression of pancreatic fibrosis in our model. Acute pancreatic damage induces the infiltration of M1 macrophages and transient activation of PSCs, while CP has prolonged activation of PSCs. A recent study showed that activated PSCs polarize infiltrated M1 macrophages towards M2, which, in turn, can more efficiently activate the PSCs [20]. This “feed-forward” process between PSCs and macrophages promotes CP-associated fibrosis. In PL pancreata, the persistent PSC activation supported by *Lats1/2* null acinar cells accelerates macrophage M2 polarization and rapidly creates a microenvironment in favor of fibrosis development. Our mouse model mimics the persistent Hippo pathway inactivation in CP. Thus, acinar cell-mediated PSC activation might be one underlying mechanism contributing to the sustained fibrosis and inflammation in CP even after the clearance of damaged tissue. A schematic showing our working model of these phenomena is shown in S11D Fig.

Although our data support that PSCs could be directly activated by secreted factors from *Lats1/2*-null acinar cells, many of these PSC activators are also immune regulators. Thus, our data do not exclude the possibility that the secreted factors released by *Lats1/2*-null acinar cells may have direct effects on immune cells. Indeed, both activated PSCs and infiltrated immune cells are found in most early lesions in *Lats1/2* knockout mice, strongly supporting that the interactions between acinar cells and immune cells may contribute to the unique phenotype observed in our model. However, understanding the detailed mechanisms of this complex acinar–immune–PSC interaction is beyond the scope of this study and may require the development of new mouse models with appropriate immune backgrounds. In addition, we observed ADM after PSC activation and immune cell infiltration. Considering the well-documented roles of inflammatory cytokines in the induction of ADM, it is unclear whether the ADM in *Lats1/2*-null pancreata is directly induced by activated YAP1/TAZ or by infiltrated immune cells. Additional mouse models are needed to answer this question.

In summary, we used adult pancreatic acinar cells as a model to investigate the physiological roles of the Hippo pathway in epithelial cells. Elegant genetic examinations enabled us to reveal a temporal pattern for epithelial Hippo disruption-induced cellular and molecular changes, which has largely been overlooked in previous studies focusing on endpoint effects of YAP1/TAZ activation. Disruption of Hippo signaling not only primes epithelial cells to exogenous pro-proliferative stimuli but also plays a critical role in activating epithelial fibroinflammatory response to signal surrounding stromal cells. These data highlight that the epithelial Hippo pathway acts as an intrinsic link between inflammatory response and proliferation during tissue repair. Experimental pancreatitis models display the molecular features associated with epithelial Hippo disruption, giving us a new perspective to understand the pathological mechanisms of these diseases and to develop effective therapies.

## Materials and methods

### Ethics statement

All procedures have been approved by Institutional Animal Care and Use Committee at the University of Texas Health San Antonio.

### Generation of conditional knockout mice

All animal study protocols were approved by the University of Texas Health San Antonio Animal Care and Use committee. *Ptf1a*^*Cre-ERTM*^ mice (The Jackson Laboratory, Bar Harbor, ME; stock number: 019378) and *R26R-EYFP* mice (The Jackson Laboratory; stock number: 006148) were obtained from Hebrok Lab [41]. *Lats1*^*fl/fl*^ and *Lats2*^*fl/fl*^ mice were kindly provided by Dr. Randy L. Johnson. *Yap1*^*fl/fl*^ and *Taz*^*fl/fl*^ mice were kindly provided by Dr. Eric N. Olson. We generated (1) *Ptf1a*^*CRE-ER*^*Rosa26*^*LSL-YFP*^ mice (P mice, control), (2) *Ptf1a*^*CRE-ER*^*Rosa26*^*LSL-YFP*^*Lats1*^*fl/fl*^*Lats2*^*fl/fl*^ *mice* (PL mice), (3) *Ptf1a*^*CRE-ER*^*Yap1*^*fl/fl*^*Taz*^*fl/fl*^ mice (PTY mice), and (4) *Ptf1a*^*CRE-ER*^*Rosa26*^*LSL-YFP*^*Lats1*^*fl/f*^*lLats2*^*fl/fl*^*Yap1*^*fl/fl*^*Taz*^*fl/fl*^ mice (PLTY mice). All offspring were genotyped by PCR of genomic DNA from the toe with primers specific for the *Ptf1a*^*Cre-ER*^, *Rosa26*^*LSL-YFP*^, *Lats1*, *Lats2*, *Yap1*, and *Taz* transgenes. To induce the conditional knockout, 6- to 12-week-old mice were treated with 45 mg/kg to 180 mg/kg of TAM (Sigma-Aldrich, St. Louis, MO, T5648-5G), which was dissolved in corn oil (Sigma-Aldrich, St. Louis, MO, C8267) via i.p. injection. PCR was used for validation of knockout alleles (S1 Table).

### Tissue preparation

Mice were euthanized, and pancreata were dissected into ice-cold phosphate-buffered saline (PBS). For histological examination, mice pancreata were fixed in 4% paraformaldehyde (PFA) overnight at 4°C and then processed for paraffin or O.C.T embedding. Sections were cut at 5 μm and then used for HE staining or immunostaining.

### Immunostaining

Primary and secondary antibodies used in this study are listed in S2 Table. For IHC staining, tissues were deparaffinized, rehydrated, and immersed and heated in a citrate-based antigen unmasking solution (Vector, Burlingame, CA, H300) for 30 minutes. Endogenous peroxidase activity was quenched with 3% hydrogen peroxide in ddH_2_O. Sections were blocked with 5% donkey serum in 0.1% TBST (Tween20, Sigma-Aldrich, St. Louis, MO, P1379-500 ml) for 1 hour at room temperature and then incubated with primary antibody at 4°C overnight. Biotin-labeled secondary antibodies (1:250, BD Pharmingen) and Streptavidin-Horseradish peroxidase (SAv-HRP, BD Pharmingen, San Jose, CA; 550946) were applied to amplify the target antigen signal, and then sections were developed with DAB substrate (Dako, Santa Clara, CA; K3468), followed by counterstaining with hematoxylin (Biocare Medical, Pacheco, CA). Finally, sections were covered with poly-Mount medium (Polysciences, Warrington, PA; Category number 08381). For immunofluorescence staining, frozen sections were blocked with 10% donkey serum plus 1% bovine serum albumin (BSA) in PBST (0.025% Triton X-100, Fisher Scientific; Waltham, MA, CAS9002-93-1) for 1 hour at room temperature. Sections were incubated with primary antibodies at 4°C overnight followed by incubation with the Alexa Fluor secondary antibodies (1:250, Jackson ImmunoResearch, West Grove, PA) for 1 hour at room temperature. Sections were covered with a drop of ProLong Gold anti-fade reagent with DAPI (Invitrogen, Carlsbad, CA, P36935) for observation. All images were obtained with a Microsystems DMI6000 B microscope and microscope software (Leica Microsystems, Buffalo Grove, IL).

### Western blot analysis

Proteins were isolated from the tail of the pancreas. Briefly, pancreata were homogenized in RIPA lysis buffer containing phosphatase and protease Inhibitors (GenDEPOT, Katy, TX; P3100-001 and P3200-001) on ice. After 10-cycle of sonication (Bioruptor Pico Sonication System, Diagenode, Denville, NJ, Category Number B01060001), proteins were extracted, and the concentration was determined by a BCA kit (Pierce, Rockford, IL; 23228). Equal amounts of 50 μg protein were resolved in SDS-PAGE, then transferred to PVDF membrane (ThermoFisher Scientific, Waltham, MA, Product number 88520) and blocked with 5% milk in PBST (0.1% Tween 20, Sigma-Aldrich, St. Louis, MO, P1379-500ml) for 1 hour at room temperature. Membranes were incubated with primary antibodies at 4°C overnight and then exposed to secondary antibodies for 1 hour. Finally, protein expression was determined by treating the membrane with Clarity Western ECL substrate (Bio-Rad, Hercules, CA, Category number 1705060) and measuring luminescence with an Amersham Imager 600 system (GE Healthcare, Waukesha, WI). (All antibody information and working concentrations are shown in S2 Table).

### RT and real-time PCR analysis

Total RNA was extracted from the tail of the pancreas using a lysis binding buffer (Ambion, Austin, TX; Category AM1560) and Direct-zol RNA miniprep kit (Zymo Research, Irvine, CA, R2052). The RNA concentration was measured using Nanodrop2000 (ThermoFisher Scientific, Waltham, MA). The amount of 400 ng of RNA was subjected to RT by using a high-capacity cDNA RT kit (Applied Biosystems; 4368814), following the manufacturer’s protocol. The cDNA samples were amplified with iTaq Universal SYBR Green Supermix (Bio-Rad, Hercules, CA; 172–5271) in a CFX96 Real-Time system (Bio-Rad, Hercules, CA). GAPDH was used as endogenous control for normalization, and the relative expression of mRNA was calculated based on the ^ΔΔ^Ct method. The information of the primers is available in S3 Table.

### Flow cytometry analysis

Single-cell solutions from P and PL mice were prepared according to previous reports. Briefly, 6-week–aged mice P and PL were treated with 180 mg/kg of TAM for 5 consecutive days. Four days later, mice were anesthetized and then perfused with cold PBS. Pancreas was digested by collagenase (Sigma-Aldrich, St. Louis, MO; C9891-1G); then, cells eventually filtrated through a 5 ml polystyrene round-bottom tube with cell-strainer cap (Corning, Corning, NY; number 352235). All antibodies used for flow cytometry were purchased from Biolegend (San Diego, CA): APC-CD45 (103111; 1:100), Percp/Cy5.5-CD11b (101228; 1:100), and BV421-F4/80 (123137; 1:100). Cells were incubated with the antibodies in FACS buffer at 4°C for 15 minutes. After incubation, cells were washed once and resuspended into FACS buffer. Sorting experiments were conducted on BD FACSAria (BD Biosciences, Franklin Lakes, NJ) with BD FACSDiva software (BD Biosciences, http://www.bdbiosciences.com/en-us/instruments/research-instruments/research-software/flow-cytometry-acquisition/facsdiva-software).

### Isolation and culture of mouse PSCs

Primary mouse PSCs were isolated using a previously reported gradient centrifugation method with a few modifications [24]. Briefly, pancreata were dissected from wild-type mice at the ages of 4 to 6 weeks. After all adipose tissues were carefully trimmed off, pancreata were cut into small pieces in cold HBSS buffer. Tissues from 2 mice were transferred into a 50 ml tube containing 10 ml HBSS buffer (0.05% pronase, 0.035% collagenase P, 0.1% DNAse, 10 mM HEPES, and 0.01 Trypsin inhibitor, Sigma-Aldrich). Tissues were incubated in a 37°C water bath for 10 minutes and then dissociated by pipetting up and down with a 10 ml tip 10 times. The tissues were incubated in a 37°C water bath for an additional 5 to 10 minutes and dispersed by pipetting gently up and down with a 10 ml pipette 10 times. The cell suspension was filtered through a 70 μm cell strainer. Cells were washed twice and suspended in 2 ml HBSS buffer containing 0.3% BSA. The amount of 4 ml 60% Optiprep was added to the cell suspension and mixed well in a 15 ml tube; 2 ml HBSS buffer was carefully added to the top of the Optiprep gradient. PSCs were separated by centrifugation at 1400*g* for 20 minutes. PSCs were suspended in DMEM:F-12 medium containing 10% FBS, 15 mM HEPES, 4 Mm glutamine, and antibiotics (penicillin 100 U/ml and streptomycin 100 μg/ml). Cells were seeded on a 10-well microscope slide (6,000 cells/well) and treated with CTGF and SPP1 for 3 days.

### Acini preparation and treatment

Immediately after euthanasia, pancreata were dissected out, rinsed in cold 1X Hank’s balanced salt solution, and cut into small pieces. The small tissue pieces were digested with collagenase P (200 μg/ml in 1X HBSS) for 20 minutes at 37 °C. Released cells were washed 3 times in cold 1X HBSS with 5% fetal bovine serum. Large clumps of connective tissues were removed by filtering with 100-μm nylon meshes. Cell suspensions were carefully layered on top of 30% fetal bovine serum for centrifuge (300*g*, 2 minutes at 4 °C) and then were resuspended in 3D culture media (RPMI, 0.5% fetal bovine serum, 0.1 mg/ml soybean trypsin inhibitor and antibiotics). Forty-eight-well tissue culture plates were coated with a 150 μl/well collagen layer (1 part 10X RPMI, 9 parts 3 mg/ml collagen, neutralized with 4.2% NaHCO_3_) and incubated for 1 hour at 37 °C. The acini suspension was mixed with collagen 1:1 and plated (0.3 ml/well). The cell–collagen mix was allowed to solidify for 1 hour at 37 °C before adding 0.5 ml 3D culture media with TGFα.

### RNA-seq and data analysis

Total RNA was isolated using RNA lysis binding buffer (Ambion, Austin, TX; Category number AM1560) at Days 1, 2, and 3, as well as control condition. RNA quality was assessed by Bioanalyzer, and poly A(+) RNA was isolated by oligo-dT purification and fragmented using divalent cations under elevated temperature. cDNA fragment libraries were synthesized following the TruSeq mRNA-seq Library Preparation protocol (Illumina). For a total of 16 samples, we obtained about 31.4 million sequence reads per sample, using Illumina HiSeq system at the Greehey Children’s Cancer Research Institute Genome Sequencing Facility, utilizing a 50 bp single-read sequencing protocol.

Short sequence reads were then aligned with TopHat [42] to mouse genome (UCSC mm^9^ https://genome.ucsc.edu/cgi-bin/hgGateway?db=mm9), allowing no more than 2 mismatches in the alignment. Expression abundance of each gene was evaluated with read count and RPKM. Differential gene expression was calculated using DESeq [43] to obtain fold-change, *P* value, and adjusted *P* value by Benjamini-Hochberg correction for multiple tests. DEGs were selected based on the following criteria: (1) fold-change > 2, (2) adjusted *P* < 0.05, and (3) RPKM > 1. Using the significance criterion and for comparison between Days 1, 2, and 3 to control, we obtained 40, 405, and 765 DEGs, respectively. To access the overall expression change across time, a heatmap was created using all DEGs from 3 comparisons. We also performed K-means algorithm (MATLAB, Mathworks) with all DEGs into 9 clusters and then merged up-regulated and down-regulated genes into 2 representative clusters (300 and 232 genes, respectively). Biological functional assessment of these DEGs was performed by using Database for Annotation, Visualization and Integrated Discovery (DAVID; http://david.abcc.ncifcrf.gov/), with the top 10 most enriched biological processes (BPs) provided in the figure.

### CTGF inhibition experiment

PL mice were injected once with neutralizing mAb FG-3019 (30 mg/kg, FibroGen) by i.p. administration. Twenty-four hours later, mice were subjected to 180 mg/kg of TAM once, and then continued to be treated with FG-3019 every other day. Human IgG (equal amount, Jackson ImmunoResearch) was used as the control. Mice were euthanized at Day 15 after TAM injection. Pancreatic tissue sections were stained with HE to quantify the severity of inflammation.

### Caerulein-induced acute and CP

Caerulein (American Peptide Company, 46-1-50) was used to induce both acute and CP mouse models. For AP, caerulein was administered through 8 i.p. injections (50 μg/kg) at hourly intervals per day for 2 days [44]. Pancreatic inflammation was assessed by HE staining on Days 1, 2, 3, 4, and 6 after induction of AP. For CP, mice were subjected to repetitive episodes of AP (50 μg/kg, 8 injections at hourly intervals) every other day for 4 weeks. Mice were euthanized 4 weeks after the initial caerulein injection.

### Statistical analysis

All data are expressed as mean ± standard error of the mean. GraphPad Prism 5 Software was used to analyze the data with ANOVA and Student *t* test.

### Transcript profiling

All RNA-seq files are available from GEO database (accession code: GSE111640).

## Supporting information

S1 Fig Acinar-specific Hippo pathway inactivation induced pancreatic inflammation-associated phenotypes in mice. (A) Mice breeding strategy and experimental design; (B) time course analysis for body weight changes of P and PL mice after 5 consecutive TAM injections (*n =* 5); (C) body weight and blood glucose levels in P and PL mice on Day 10 after final TAM injection; underlying numerical values can be found in S1 Data. (D) Time course HE analysis for pancreata of PL mice with 5 consecutive TAM injections.
(TIF)

S2 Fig YAP and TAZ activations were regulated by Lats1/2 in adult acinar cells. (A) Scheme of the mouse *Lats1* and *Lats2* locus and the strategy of detecting *Lats1/2* deletions. Confirmation of the excisions of *Lats1* exon 4 (deletion: 230 bp) and *Lats2* exon 5 (deletion: 250 bp) in PL mouse by PCR. The primer sequences are indicated as P1, P2, and P3 for *Lats1* detection and P1’, P2’, and P3’ for *Lats2* detection; (B) *Lats1/2*-deletion–induced YAP1/TAZ translocation was detected by IHC staining in P, PL, PL1KO, and PL2KO mice (*n =* 6).
(TIF)

S3 Fig Acinar-specific Lats1/2 depletions induced pancreatitis-associated histological alterations. (A) ADM was quantified by counting YFP and CK19 double-positive cell numbers. CD45 and αSMA were quantified by IHC profiler score (*n =* 5). *P < 0.05, **P < 0.01. Representative immunofluorescence staining with (B) anti-YFP (Green), anti-CK19 (Red), anti-Ki67 (White) antibodies and with (C) anti-YFP (Green), anti-CK19 (Red), anti-cleaved-caspase-3 (White) antibodies in P and PL pancreata. Nuclei stained with DAPI (Blue). Ki67 and cleaved-caspase-3 were quantified by relative fluorescence (*n =* 5); **P < 0.01. Underlying numerical values can be found in S1 Data.
(TIF)

S4 Fig Generation of mice with *Lats1*, *Lats2*, *Yap1*, *Taz* quadruple deletions in pancreatic acinar cells. (A) Generation of PTY mice and the strategy for detecting *Yap1*/*Taz* deletion. HE staining was performed in P and PTY mice; (B) PLTY mice breeding strategy and experimental design; (C) quantification of western blot of LATS1, LATS2, YAP1, and TAZ in PL and PLTY mice. P mice served as the control group. Tubulin was used as the internal control (*n =* 6); **P < 0.01. Underlying numerical values can be found in S1 Data.
(TIF)

S5 Fig Mosaic Lats1/2 deletion induced long-lasting pancreatic inflammation. (A) PL mice were injected once with 45 mg/kg, 90 mg/kg, or 180 mg/kg of TAM, respectively. Confirmation of the excisions of *Lats1* exon 4 and *Lats2* exon 5 by PCR at 45 mg/kg of TAM condition. *Lats1* deletion: 230 bp; *Lats2* deletion: 250 bp. (B) Anti-YFP antibody (Green) was used to stain *Lats1/2* null cells 2 days later. Nuclei stained with DAPI (Blue). (C) Three weeks later, mice among injection groups were euthanized, and pancreata were stained with HE, anti-CD45, anti-αSMA, and anti-CK19 antibodies (*n =* 4). (D) YFP+ and YFP− cells were sorted by flow cytometry from PL mice 8 days after one-time 45 mg/kg TAM injection. Excision of *LATS1* exon 4 and *LATS2* exon 5 in YFP+ cells was confirmed by PCR. (E) P and PL mice were consecutively injected with 5 doses (180 mg/kg) of TAM. Primary pancreatic acini were isolated 3 days after final injection and embedded into collagen for 3D culture (*n =* 3). Cells were treated with or without TGFα (100 ng/mL) for 5 days.
(TIF)

S6 Fig Effect of Lats1/2 knockout on ADM, PSC activation, and immune cell infiltration. (A) Time course quantification of ADM, PSC activation, and immune cell infiltration in the pancreas of PL mice after a single-dose TAM injection (180 mg/kg) (*n =* 4). Underlying numerical values can be found in S1 Data. (B) PL mice were injected once with 180 mg/kg of TAM. ADM, PSC activation, and immune cell infiltration were detected by anti-CK19, anti-αSMA, and anti-CD45 antibodies on Day 10 and Day 20 after TAM injection.
(TIF)

S7 Fig Examine the effects of Lats1/2 deletions on macrophage polarizations. (A) Time course analysis of immune cell infiltration in the pancreas of P and PL mice after 5 consecutive TAM injections. Immune cells were stained with anti-CD45 antibody (*n =* 3). (B) Gating strategy to sort macrophages for quantitative RT-PCR assay. Immune cells were stained with CD45 (P1: red). CD45^+^CD11b^+^F4/80^+^ macrophages were sorted (P2: blue).
(TIF)

S8 Fig Lats1/2 deletions in pancreatic acinar cells induce CP-like phenotype rapidly and SPP1 is strongly associated with PSC activation. (A) HE staining of PL mice after TAM injection of 180 mg/kg/day for 5 consecutive days via i.p. *n =* 4. (B) αSMA, CK19, and CD45 IHC staining in consecutive sections at Day 2 and Day 3 after final injection. (C) The mRNA expression of Lats1, Lats2, Ctgf, Cyr61, and Spp1 were measured by qPCR in P and PL (D2) mice. **P < 0.01. Underlying numerical values can be found in S1 Data. (D) Small lesion was co-stained with αSMA (Red) and SPP1 (Green) in PL mice (180 mg/kg of TAM, Day 10) by immunofluorescence. Nuclei stained with DAPI (Blue).
(TIF)

S9 Fig Examination of the effects of CTGF and SPP1 on PSC activation in vitro. (A) Representative immunofluorescent staining of GFAP in isolated mouse PSCs. (B) The small lesions were stained with anti-αSMA, anti-SPP1, anti-YAP1, and anti-TAZ antibodies in consecutive sections. (C) Immunofluorescent staining for SPP1 in P, PL, and PLTY pancreata 5 days after injection of 5 times of 180 mg/kg TAM. (D) mRNA levels of SPP1 in P, PL, and PLTY pancreata 5 days after injection of 5 times of 180 mg/kg TAM as determined by RT-PCR (*n =* 4). **P < 0.01. Underlying numerical values can be found in S1 Data.
(TIF)

S10 Fig Depletion of CTGF with neutralization antibody to rescue Lats1/2 knockout-induced phenotypes. (A) Quantification of western blot of CTGF in PL and PLTY mice. Tubulin was used as the internal control (*n =* 6); **P < 0.01. (B) Effects of FG-3019 treatment on body weights of PL mice that received TAM injection. Mice injected with FG-huIgG antibody were used as the control (*n =* 5–6). Underlying numerical values can be found in S1 Data.
(TIF)

S11 Fig Hippo pathway is dynamically regulated in pancreatitis models. (A) Quantification of western blot of LATS1, LATS2, P-YAP1, YAP1, and TAZ in untreated and AP mice. Untreated mice served as the control group. Tubulin was used as the internal control (*n =* 3); (B) immunochemistry staining to detect YAP1 expression on Day 1 after AP induction (*n =* 4); (C) quantification of western blot of LATS1, LATS2, YAP1, and TAZ in untreated and CP mice. Untreated mice served as the control group. Tubulin was used as the internal control (*n =* 3). **P < 0.01. Underlying numerical values can be found in S1 Data. (D) Schematic of the working model of the effect of the Hippo pathway in pancreatic acinar cells. Hippo pathway inactivation-induced YAP1/TAZ nuclear translocation activates the fibroinflammatory transcriptional program in adult acinar cells, leading to up-regulation of secretary factors such as CTGF and SPP1 in acinar cells. These factors activate surrounding PSCs and immune cells, which might provide feedback signals to promote acinar proliferation and ADM. Permanent Hippo pathway inactivation in acinar cells triggers persistent PSC activation, contributing to the development of pancreatic fibrosis.
(TIF)

S1 Table Primer sequences used for genotyping. (DOCX)

S2 Table List of antibodies used in the study. (DOCX)

S3 Table Primer sequences used for real-time PCR in the study. (DOCX)

S1 Data Numerical data used in Figs 2, 3, 5, 6, 7, S1, S3, S4, S6, S8, S9, S10, and S11. (XLSX)

S1 Raw Images Raw images used in Figs 1, 2, 7, and 8. (PDF)

## Abbreviations

αSMA: α-smooth muscle actin
ADM: acinar-to-ductal metaplasia
AP: acute pancreatitis
BP: biological process
BSA: bovine serum albumin
CD45: cluster of differentiation antigen 45
CK19: cytokeratin 19
CP: chronic pancreatitis
CTGF: connective tissue growth factor
DAVID: Database for Annotation, Visualization and Integrated Discovery
DEG: differentially expressed gene
ECM: extracellular matrix
EdU: 5-ethynyl-2'-deoxyuridine
GFAP: Glial fibrillary acidic protein
GO: gene ontology
HE: hematoxylin–eosin
HSC: hepatic stellate cell
IHC: immunohistochemistry
i.p.: intraperitoneal
Ihh: Indian hedgehog
Ki67: antigen identified by monoclonal antibody Ki 67
LATS1: large tumor suppressor 1
NR5A2: nuclear receptor subfamily 5, group A, member 2
P-YAP1: phospho-YAP1
PBS: phosphate-buffered saline
PFA: paraformaldehyde
PSC: pancreatic stellate cell
qPCR: quantitative PCR
RNA-seq: RNA sequencing
RPKM: read per kilobase of transcript per million reads mapped
RT: reverse transcription
SPP1: secreted phosphoprotein 1
TAM: tamoxifen
TAZ: transcriptional coactivator with PDZ binding motif
TGFβ: transforming growth factor, beta
Yap1: yes-associated protein 1
YFP: yellow fluorescent protein
