# Supplementary figures and images for: Activation of the intrinsic fibroinflammatory program in adult pancreatic acinar cells triggered by Hippo signaling disruption
Source: PLoS Biol. 2019 Sep 12;17(9):e3000418. doi: 10.1371/journal.pbio.3000418 (PMC6742234; doi:10.1371/journal.pbio.3000418)

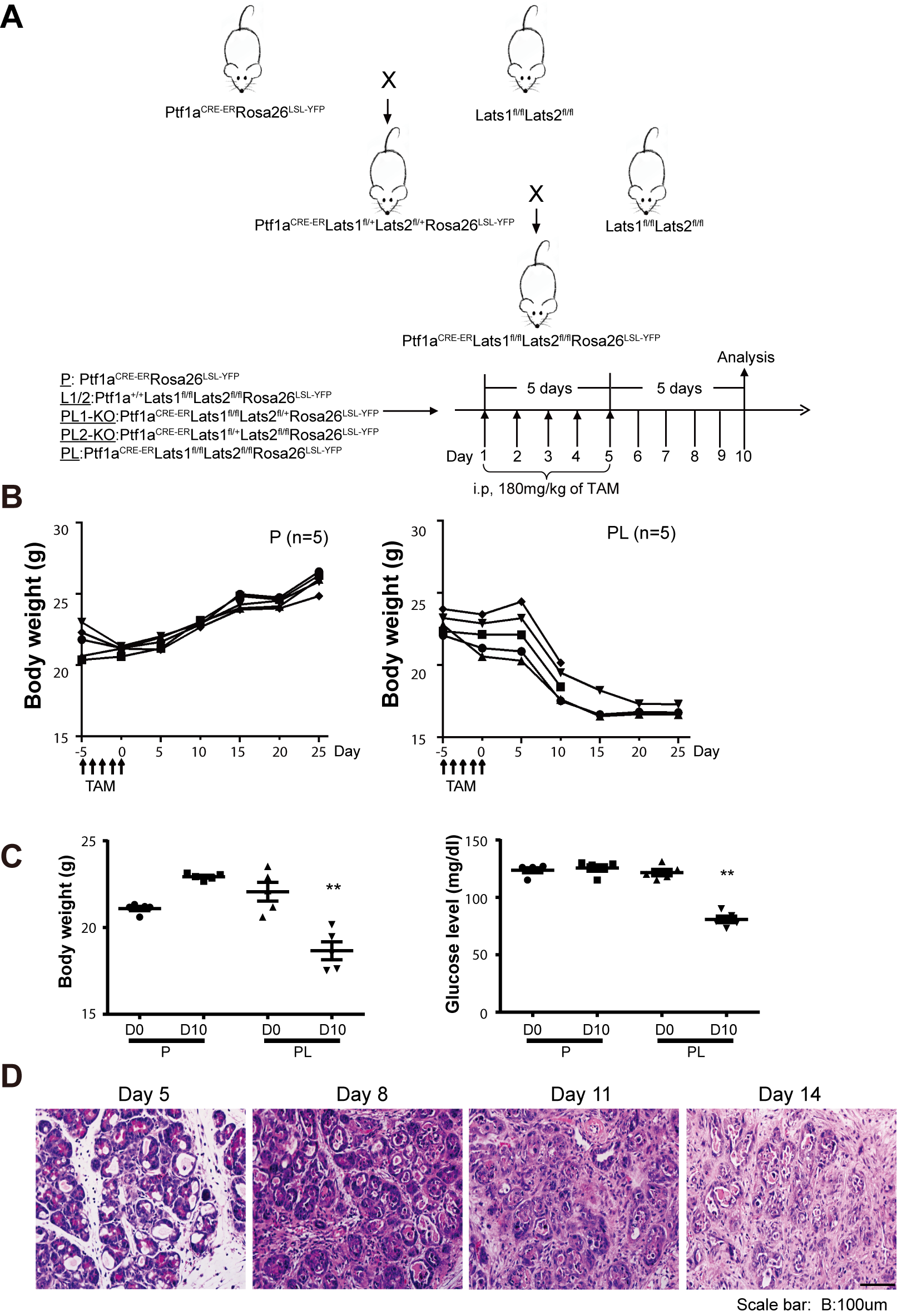

Supplement: S1 Fig — (A) Mice breeding strategy and experimental design; (B) time course analysis for body weight changes of P and PL mice after 5 consecutive TAM injections (n = 5); (C) body weight and blood glucose levels in P and PL mice on Day 10 after final TAM injection; underlying numerical values can be found in S1 Data. (D) Time course HE analysis for pancreata of PL mice with 5 consecutive TAM injections. (TIF) [file pbio.3000418.s001.tif]

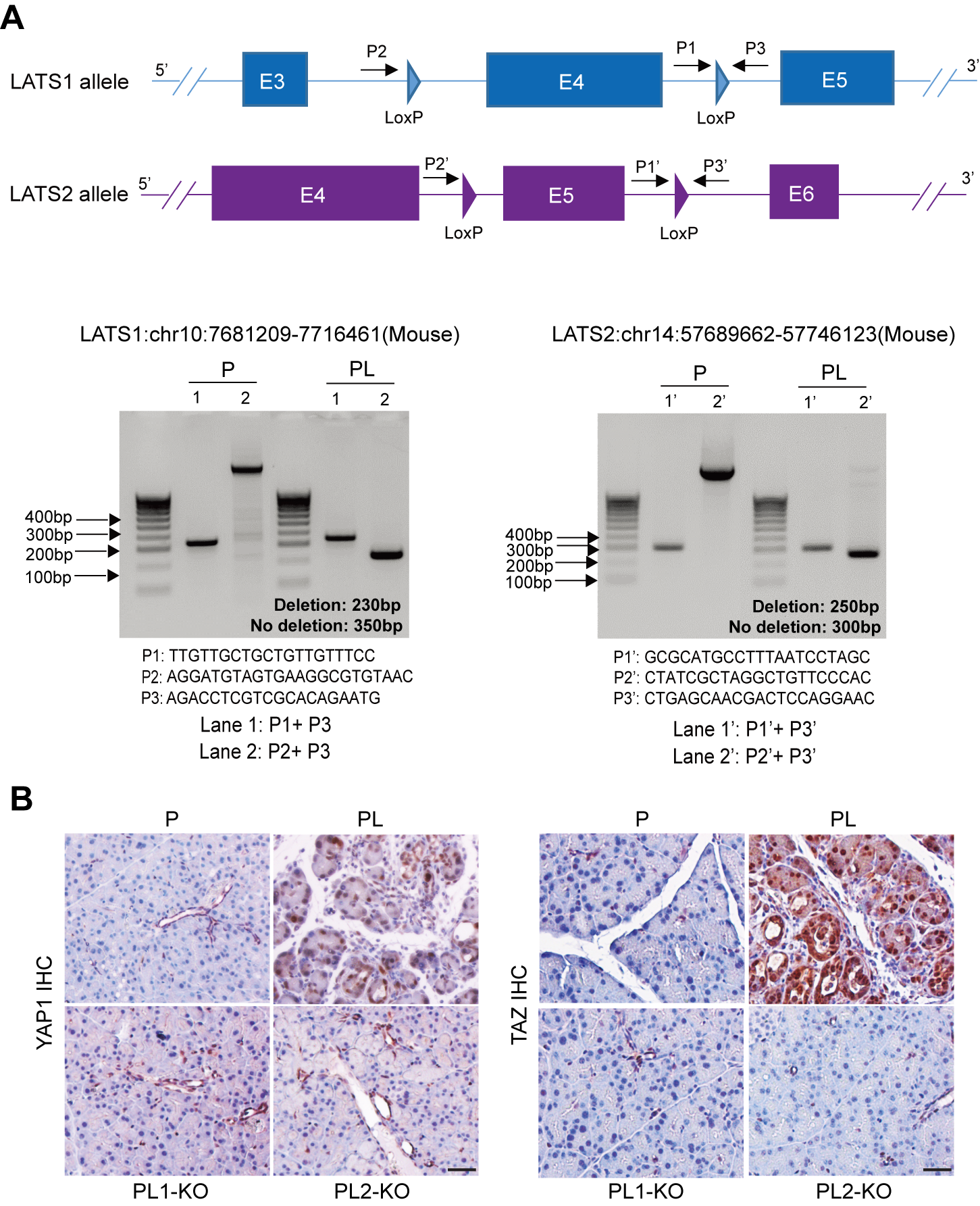

Supplement: S2 Fig — (A) Scheme of the mouse Lats1 and Lats2 locus and the strategy of detecting Lats1/2 deletions. Confirmation of the excisions of Lats1 exon 4 (deletion: 230 bp) and Lats2 exon 5 (deletion: 250 bp) in PL mouse by PCR. The primer sequences are indicated as P1, P2, and P3 for Lats1 detection and P1’, P2’, and P3’ for Lats2 detection; (B) Lats1/2-deletion–induced YAP1/TAZ translocation was detected by IHC staining in P, PL, PL1KO, and PL2KO mice (n = 6). (TIF) [file pbio.3000418.s002.tif]

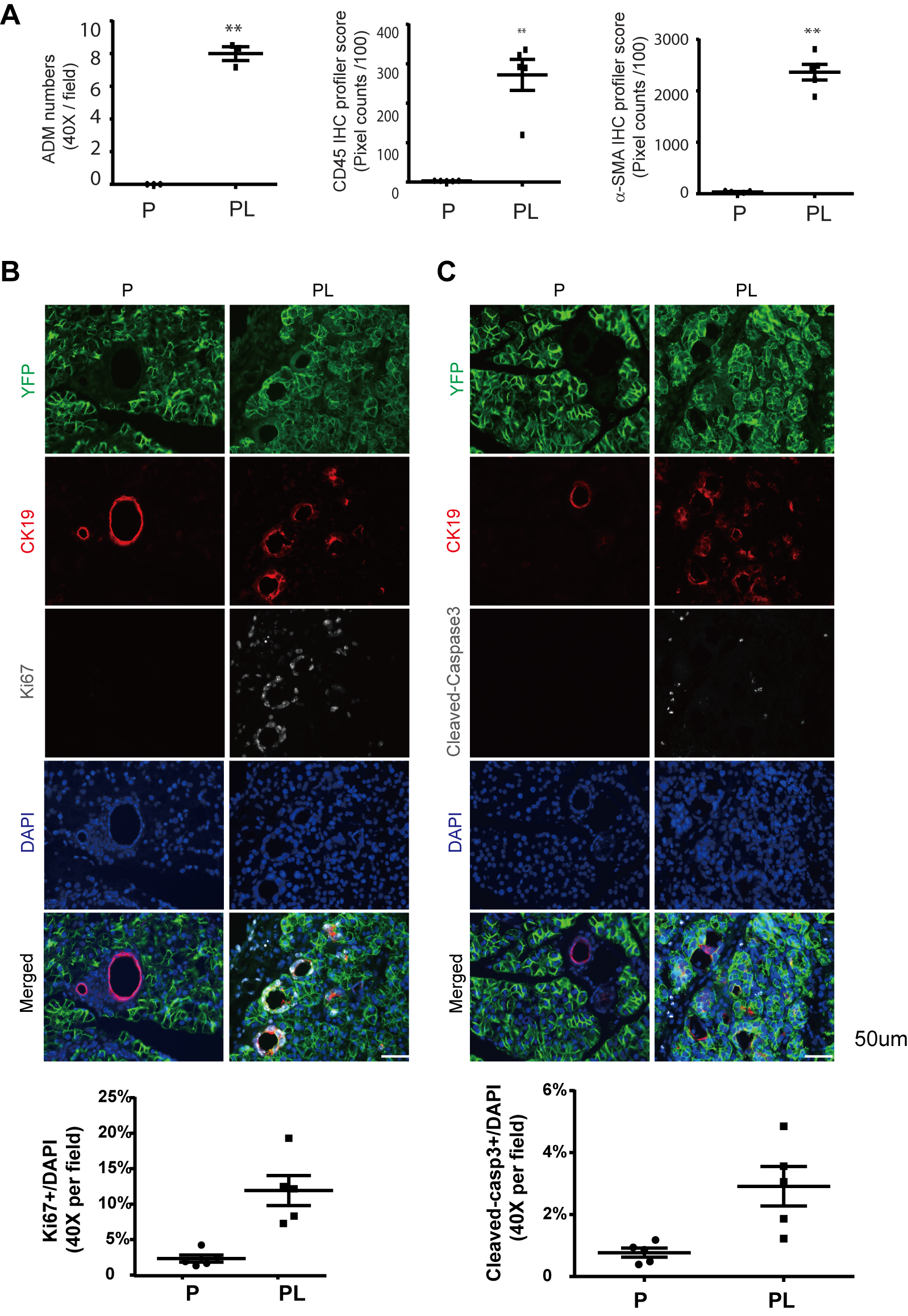

Supplement: S3 Fig — (A) ADM was quantified by counting YFP and CK19 double-positive cell numbers. CD45 and αSMA were quantified by IHC profiler score (n = 5). *P < 0.05, **P < 0.01. Representative immunofluorescence staining with (B) anti-YFP (Green), anti-CK19 (Red), anti-Ki67 (White) antibodies and with (C) anti-YFP (Green), anti-CK19 (Red), anti-cleaved-caspase-3 (White) antibodies in P and PL pancreata. Nuclei stained with DAPI (Blue). Ki67 and cleaved-caspase-3 were quantified by relative fluorescence (n = 5); **P < 0.01. Underlying numerical values can be found in S1 Data. (TIF) [file pbio.3000418.s003.tif]

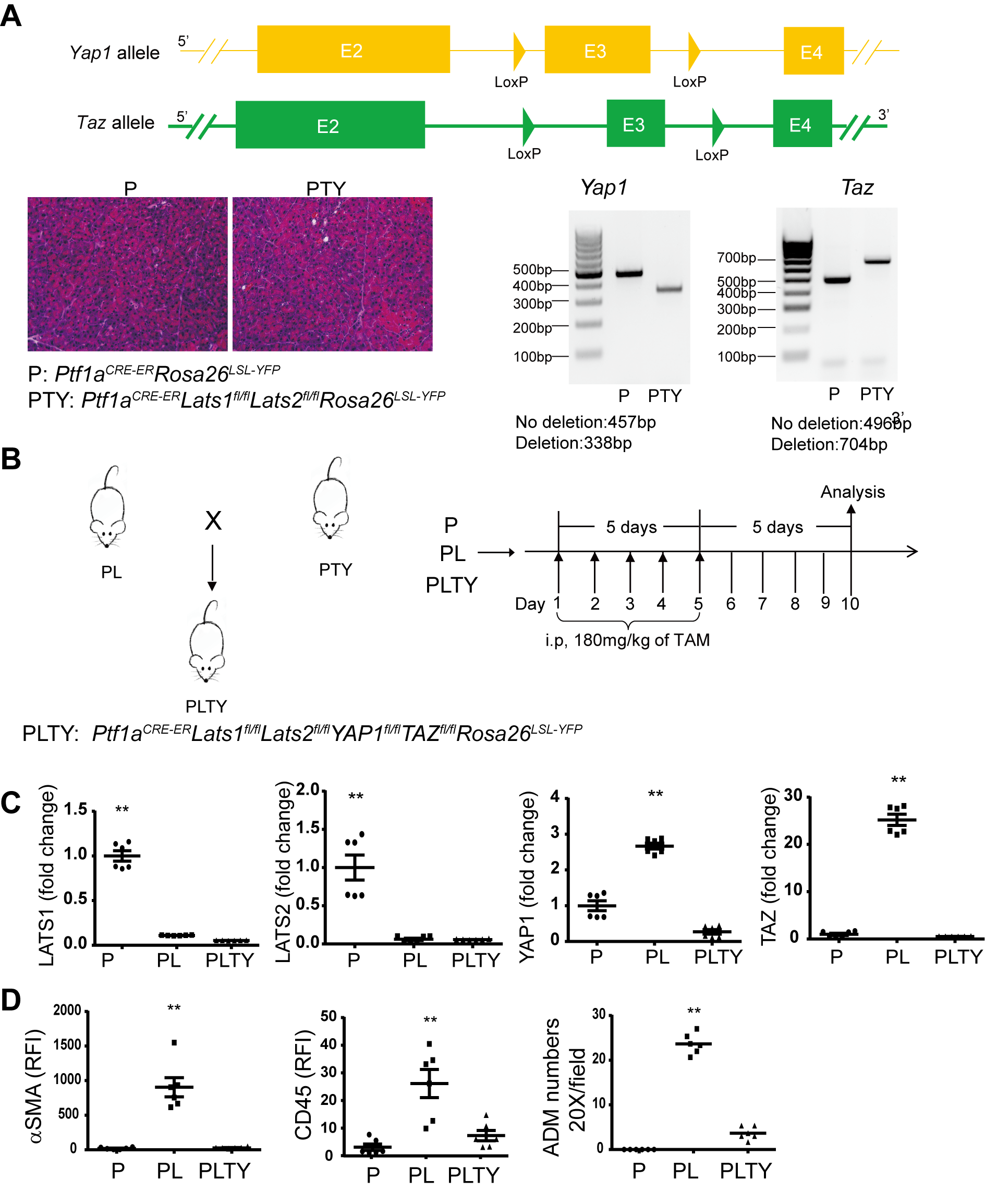

Supplement: S4 Fig — (A) Generation of PTY mice and the strategy for detecting Yap1/Taz deletion. HE staining was performed in P and PTY mice; (B) PLTY mice breeding strategy and experimental design; (C) quantification of western blot of LATS1, LATS2, YAP1, and TAZ in PL and PLTY mice. P mice served as the control group. Tubulin was used as the internal control (n = 6); **P < 0.01. Underlying numerical values can be found in S1 Data. (TIF) [file pbio.3000418.s004.tif]

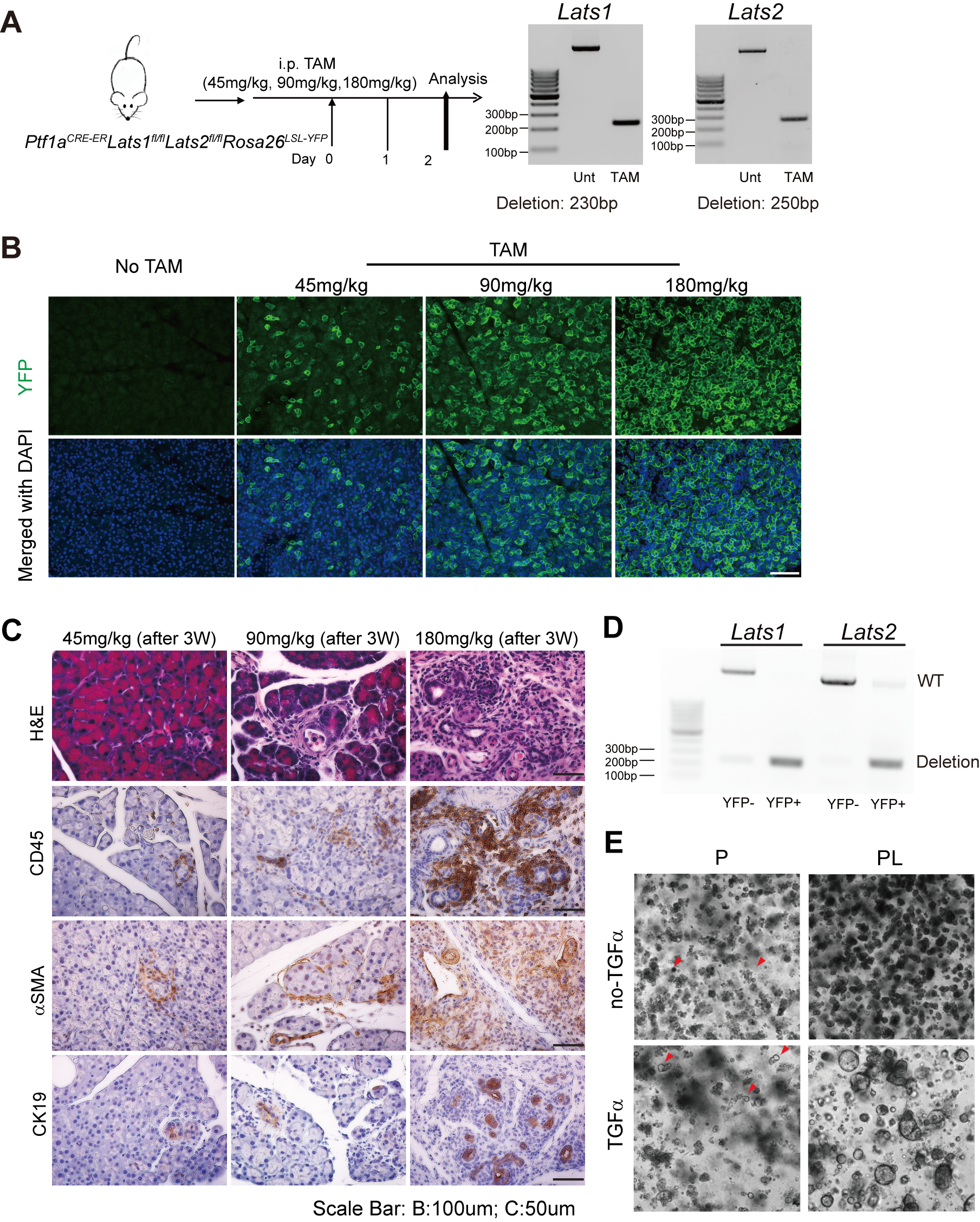

Supplement: S5 Fig — (A) PL mice were injected once with 45 mg/kg, 90 mg/kg, or 180 mg/kg of TAM, respectively. Confirmation of the excisions of Lats1 exon 4 and Lats2 exon 5 by PCR at 45 mg/kg of TAM condition. Lats1 deletion: 230 bp; Lats2 deletion: 250 bp. (B) Anti-YFP antibody (Green) was used to stain Lats1/2 null cells 2 days later. Nuclei stained with DAPI (Blue). (C) Three weeks later, mice among injection groups were euthanized, and pancreata were stained with HE, anti-CD45, anti-αSMA, and anti-CK19 antibodies (n = 4). (D) YFP+ and YFP− cells were sorted by flow cytometry from PL mice 8 days after one-time 45 mg/kg TAM injection. Excision of LATS1 exon 4 and LATS2 exon 5 in YFP+ cells was confirmed by PCR. (E) P and PL mice were consecutively injected with 5 doses (180 mg/kg) of TAM. Primary pancreatic acini were isolated 3 days after final injection and embedded into collagen for 3D culture (n = 3). Cells were treated with or without TGFα (100 ng/mL) for 5 days. (TIF) [file pbio.3000418.s005.tif]

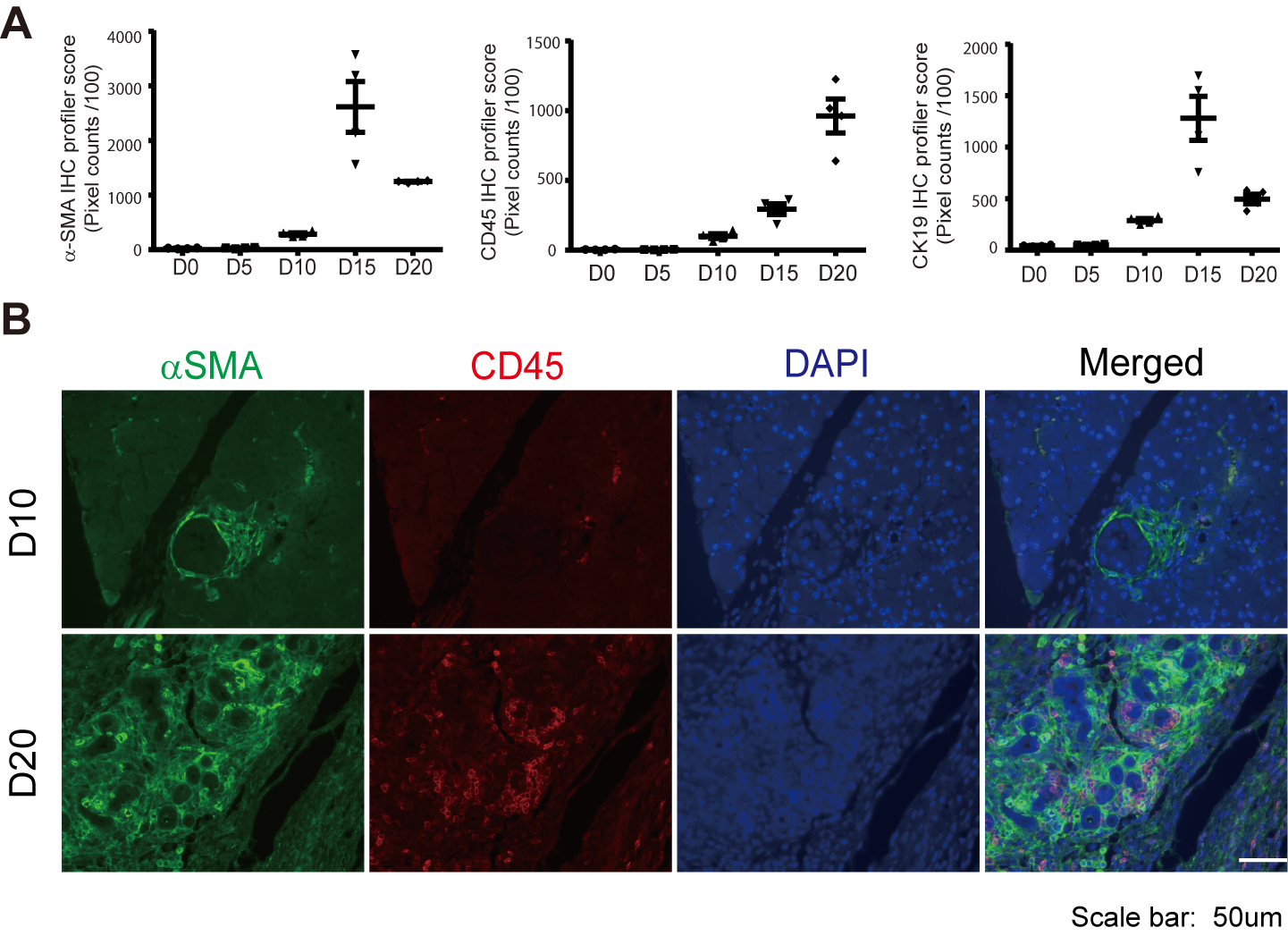

Supplement: S6 Fig — (A) Time course quantification of ADM, PSC activation, and immune cell infiltration in the pancreas of PL mice after a single-dose TAM injection (180 mg/kg) (n = 4). Underlying numerical values can be found in S1 Data. (B) PL mice were injected once with 180 mg/kg of TAM. ADM, PSC activation, and immune cell infiltration were detected by anti-CK19, anti-αSMA, and anti-CD45 antibodies on Day 10 and Day 20 after TAM injection. (TIF) [file pbio.3000418.s006.tif]

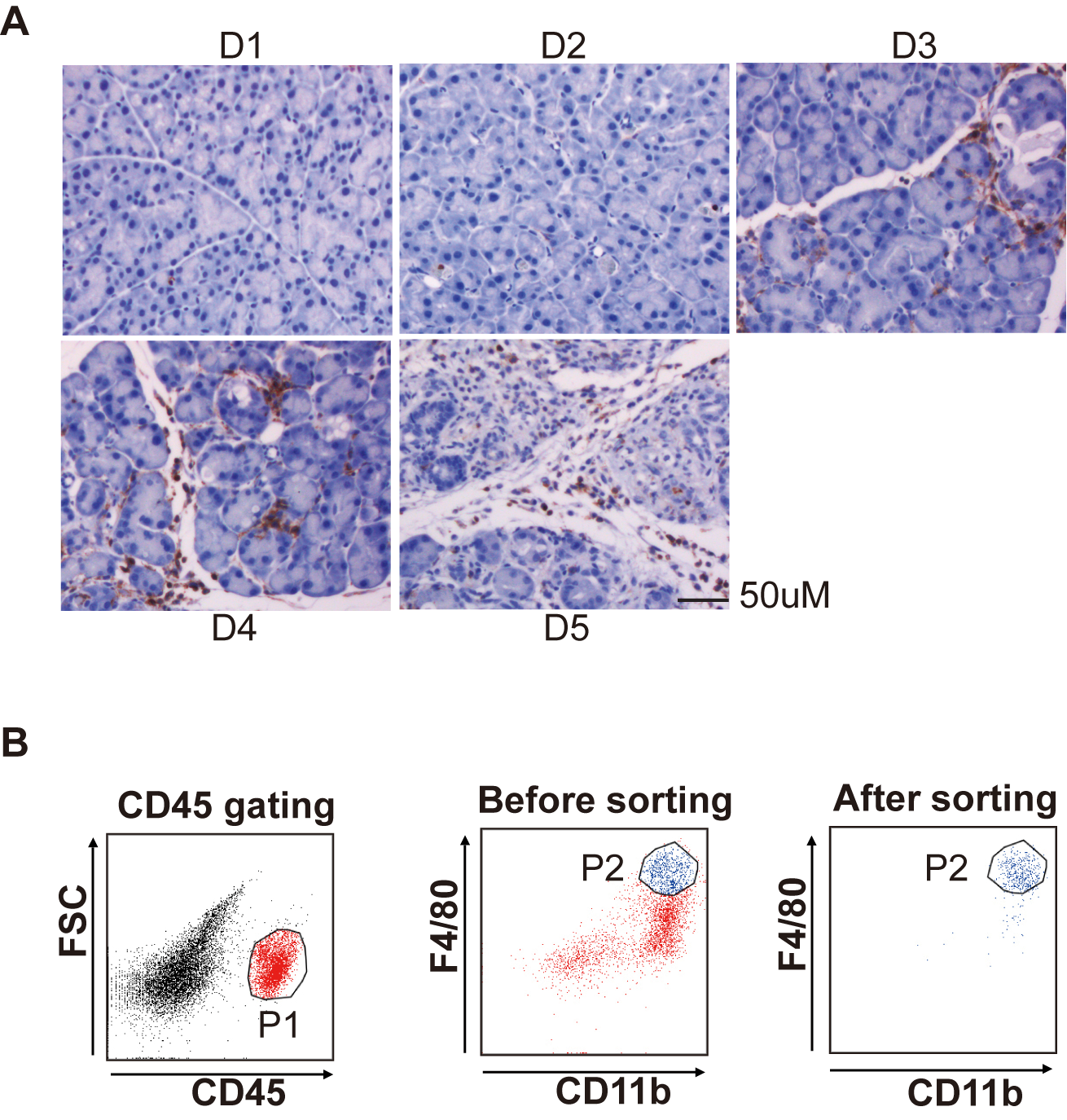

Supplement: S7 Fig — (A) Time course analysis of immune cell infiltration in the pancreas of P and PL mice after 5 consecutive TAM injections. Immune cells were stained with anti-CD45 antibody (n = 3). (B) Gating strategy to sort macrophages for quantitative RT-PCR assay. Immune cells were stained with CD45 (P1: red). CD45+CD11b+F4/80+ macrophages were sorted (P2: blue). (TIF) [file pbio.3000418.s007.tif]

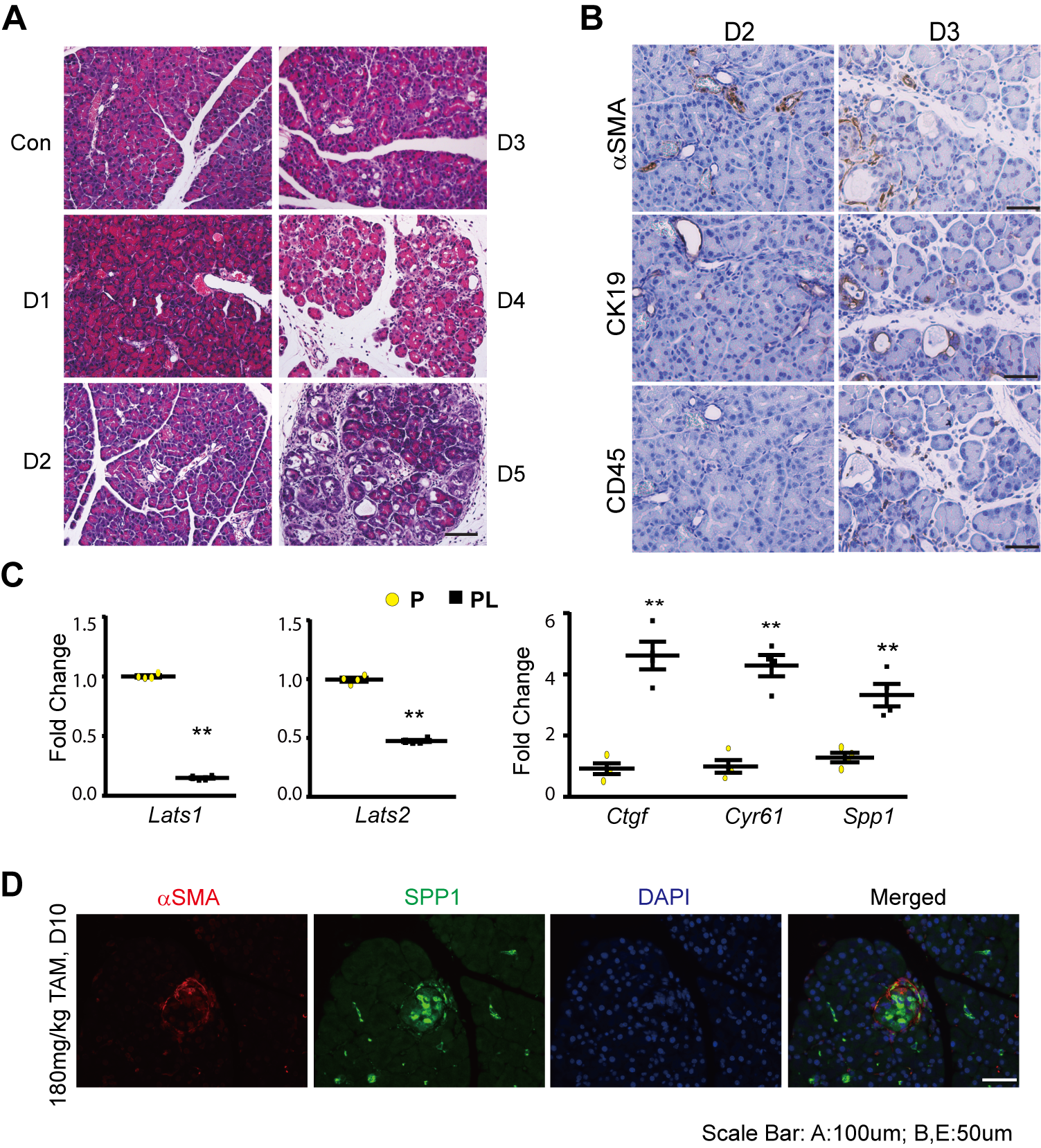

Supplement: S8 Fig — (A) HE staining of PL mice after TAM injection of 180 mg/kg/day for 5 consecutive days via i.p. n = 4. (B) αSMA, CK19, and CD45 IHC staining in consecutive sections at Day 2 and Day 3 after final injection. (C) The mRNA expression of Lats1, Lats2, Ctgf, Cyr61, and Spp1 were measured by qPCR in P and PL (D2) mice. **P < 0.01. Underlying numerical values can be found in S1 Data. (D) Small lesion was co-stained with αSMA (Red) and SPP1 (Green) in PL mice (180 mg/kg of TAM, Day 10) by immunofluorescence. Nuclei stained with DAPI (Blue). (TIF) [file pbio.3000418.s008.tif]

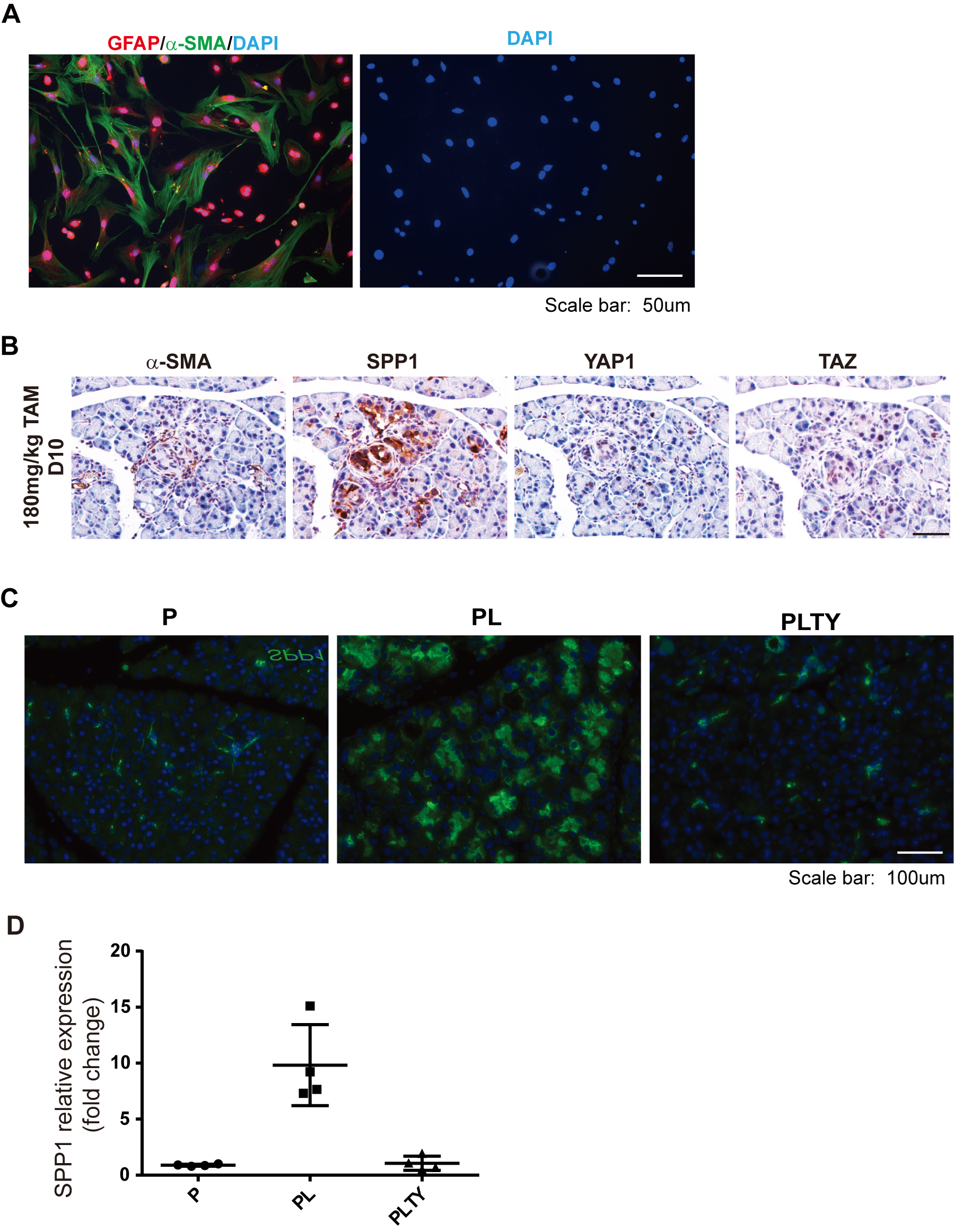

Supplement: S9 Fig — (A) Representative immunofluorescent staining of GFAP in isolated mouse PSCs. (B) The small lesions were stained with anti-αSMA, anti-SPP1, anti-YAP1, and anti-TAZ antibodies in consecutive sections. (C) Immunofluorescent staining for SPP1 in P, PL, and PLTY pancreata 5 days after injection of 5 times of 180 mg/kg TAM. (D) mRNA levels of SPP1 in P, PL, and PLTY pancreata 5 days after injection of 5 times of 180 mg/kg TAM as determined by RT-PCR (n = 4). **P < 0.01. Underlying numerical values can be found in S1 Data. (TIF) [file pbio.3000418.s009.tif]

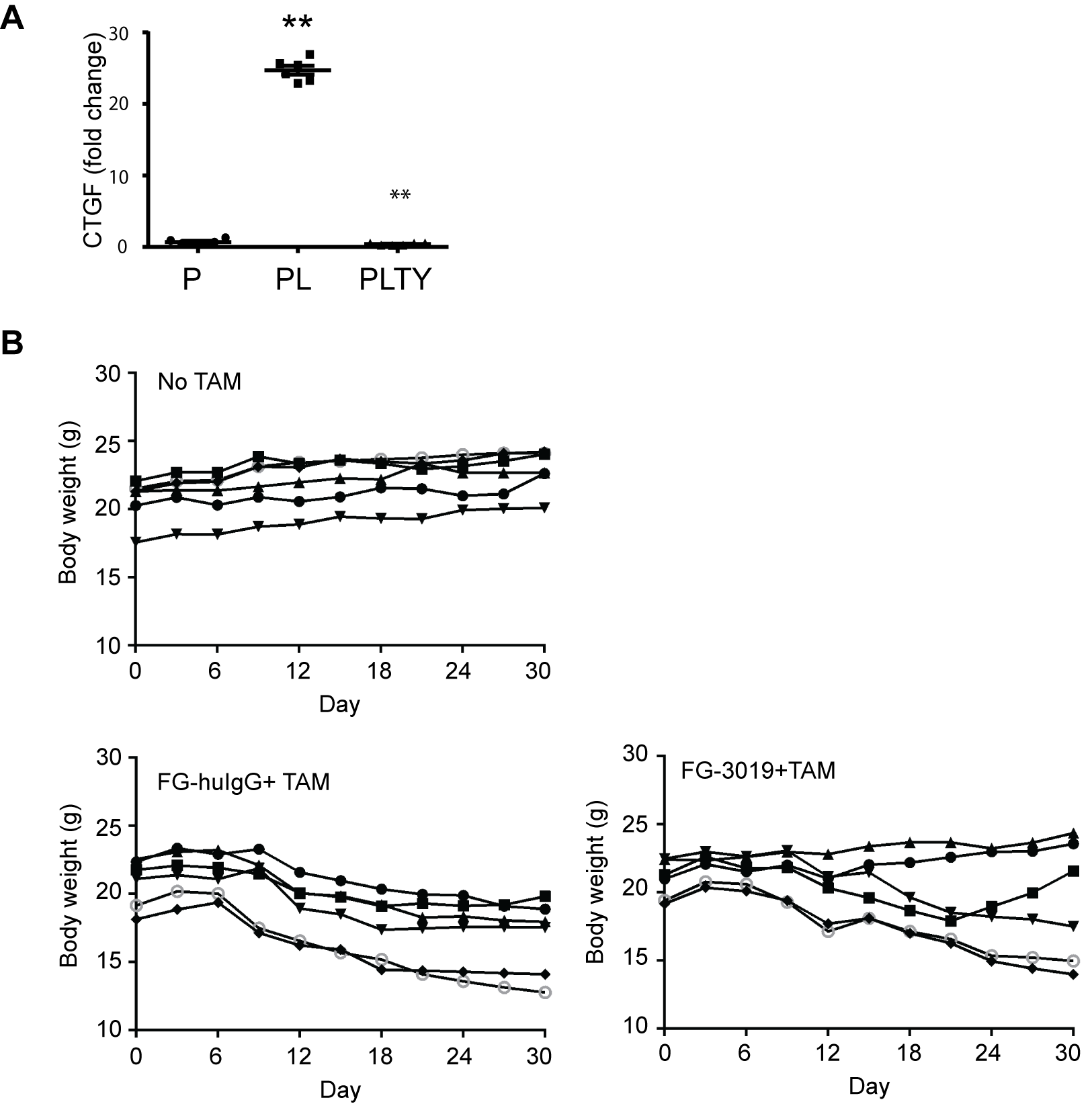

Supplement: S10 Fig — (A) Quantification of western blot of CTGF in PL and PLTY mice. Tubulin was used as the internal control (n = 6); **P < 0.01. (B) Effects of FG-3019 treatment on body weights of PL mice that received TAM injection. Mice injected with FG-huIgG antibody were used as the control (n = 5–6). Underlying numerical values can be found in S1 Data. (TIF) [file pbio.3000418.s010.tif]

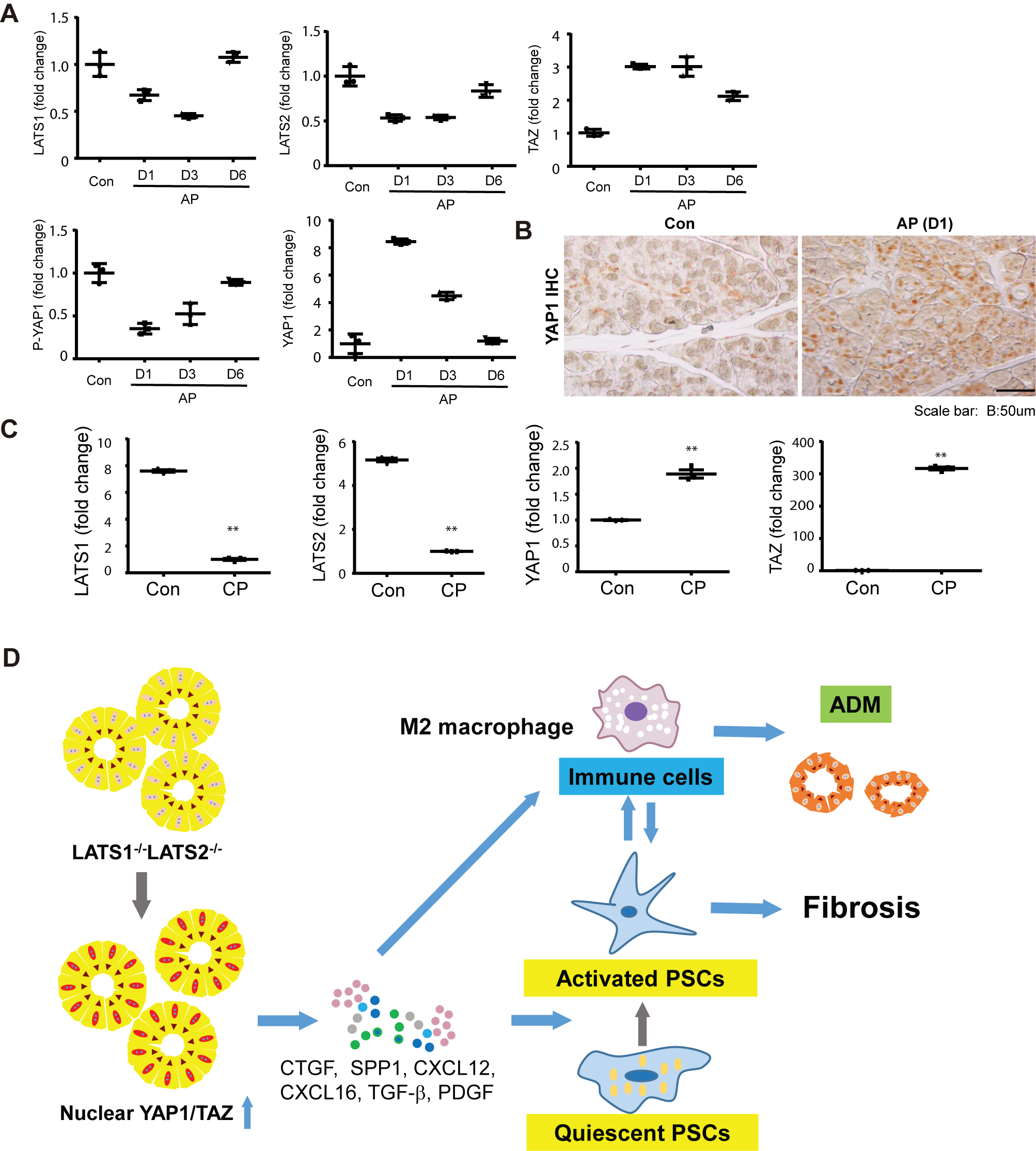

Supplement: S11 Fig — (A) Quantification of western blot of LATS1, LATS2, P-YAP1, YAP1, and TAZ in untreated and AP mice. Untreated mice served as the control group. Tubulin was used as the internal control (n = 3); (B) immunochemistry staining to detect YAP1 expression on Day 1 after AP induction (n = 4); (C) quantification of western blot of LATS1, LATS2, YAP1, and TAZ in untreated and CP mice. Untreated mice served as the control group. Tubulin was used as the internal control (n = 3). **P < 0.01. Underlying numerical values can be found in S1 Data. (D) Schematic of the working model of the effect of the Hippo pathway in pancreatic acinar cells. Hippo pathway inactivation-induced YAP1/TAZ nuclear translocation activates the fibroinflammatory transcriptional program in adult acinar cells, leading to up-regulation of secretary factors such as CTGF and SPP1 in acinar cells. These factors activate surrounding PSCs and immune cells, which might provide feedback signals to promote acinar proliferation and ADM. Permanent Hippo pathway inactivation in acinar cells triggers persistent PSC activation, contributing to the development of pancreatic fibrosis. (TIF) [file pbio.3000418.s011.tif]

Fig 1B

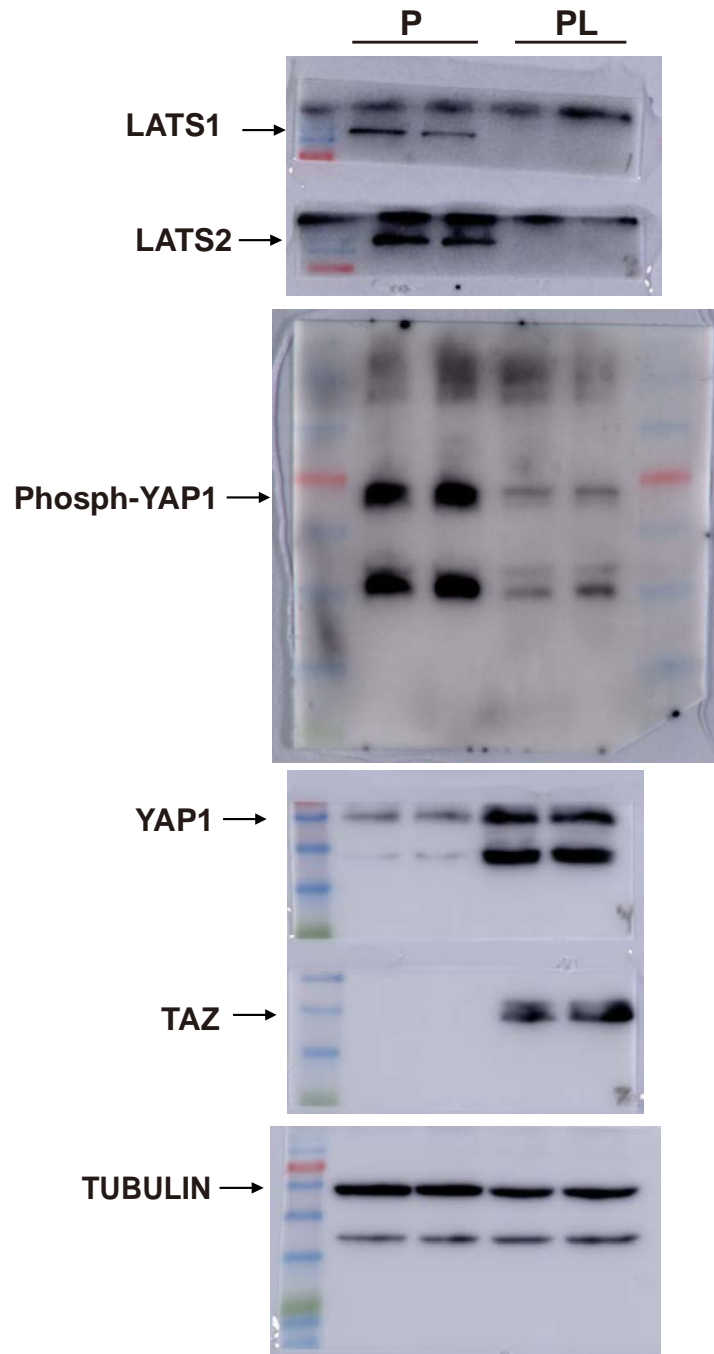

Fig 2A

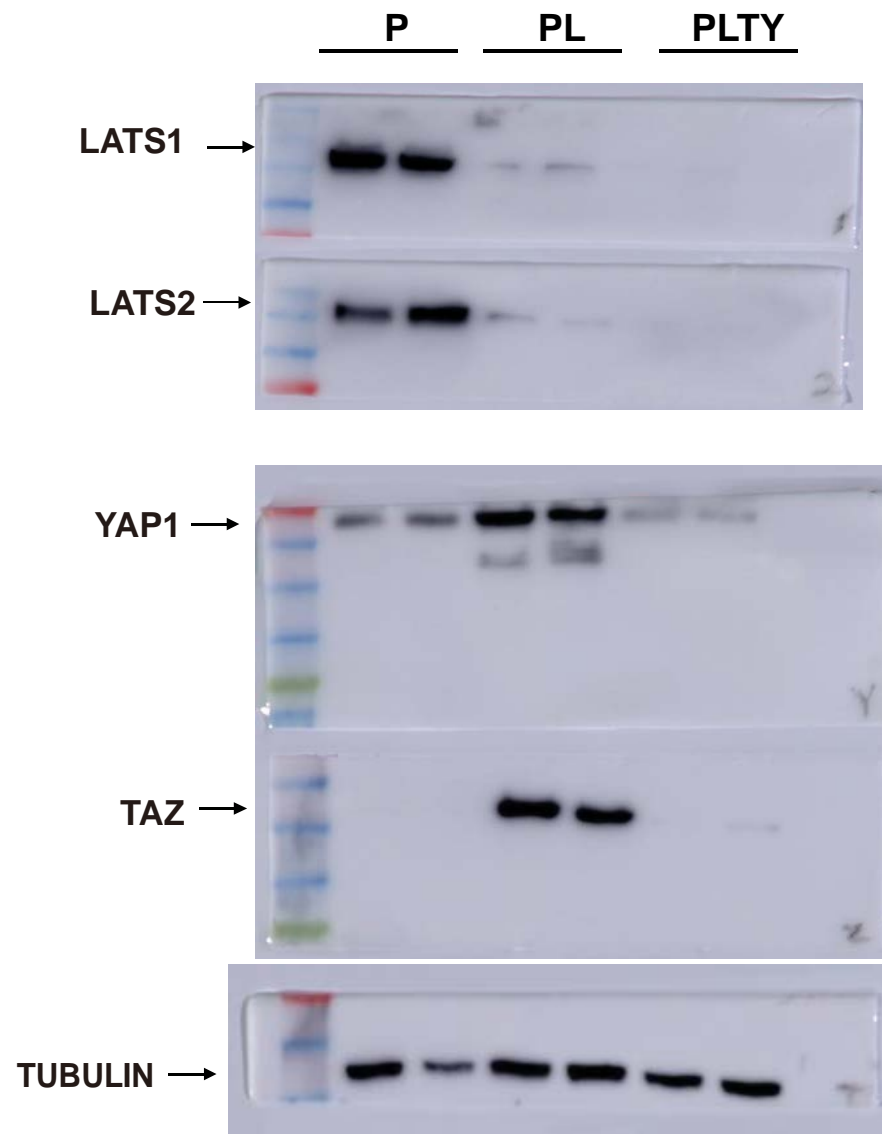

Fig 7B

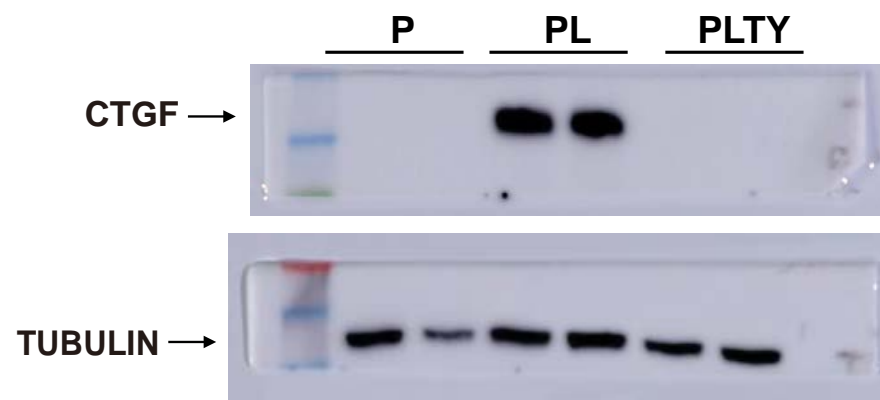

Fig 8C

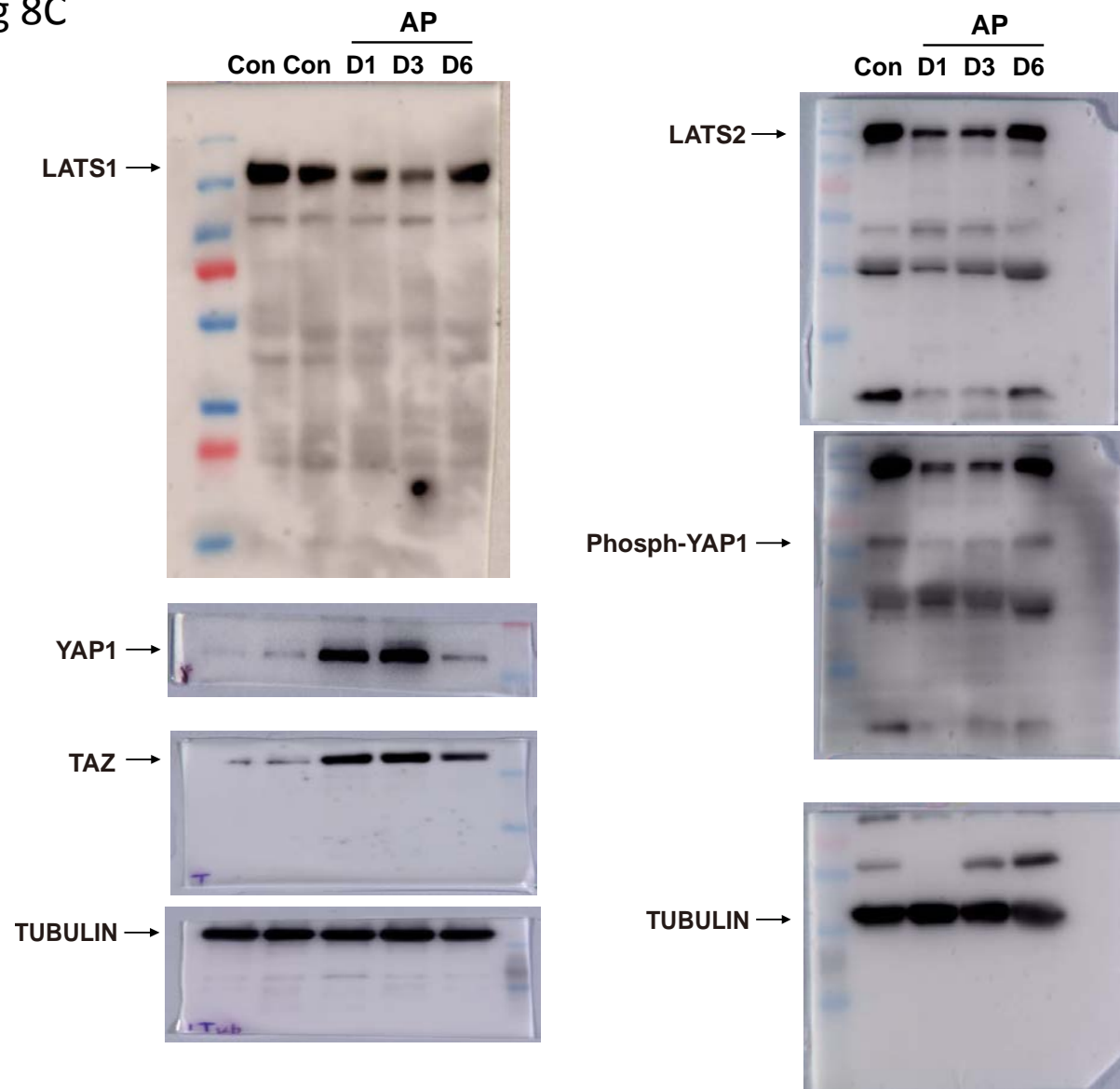

Fig 8F

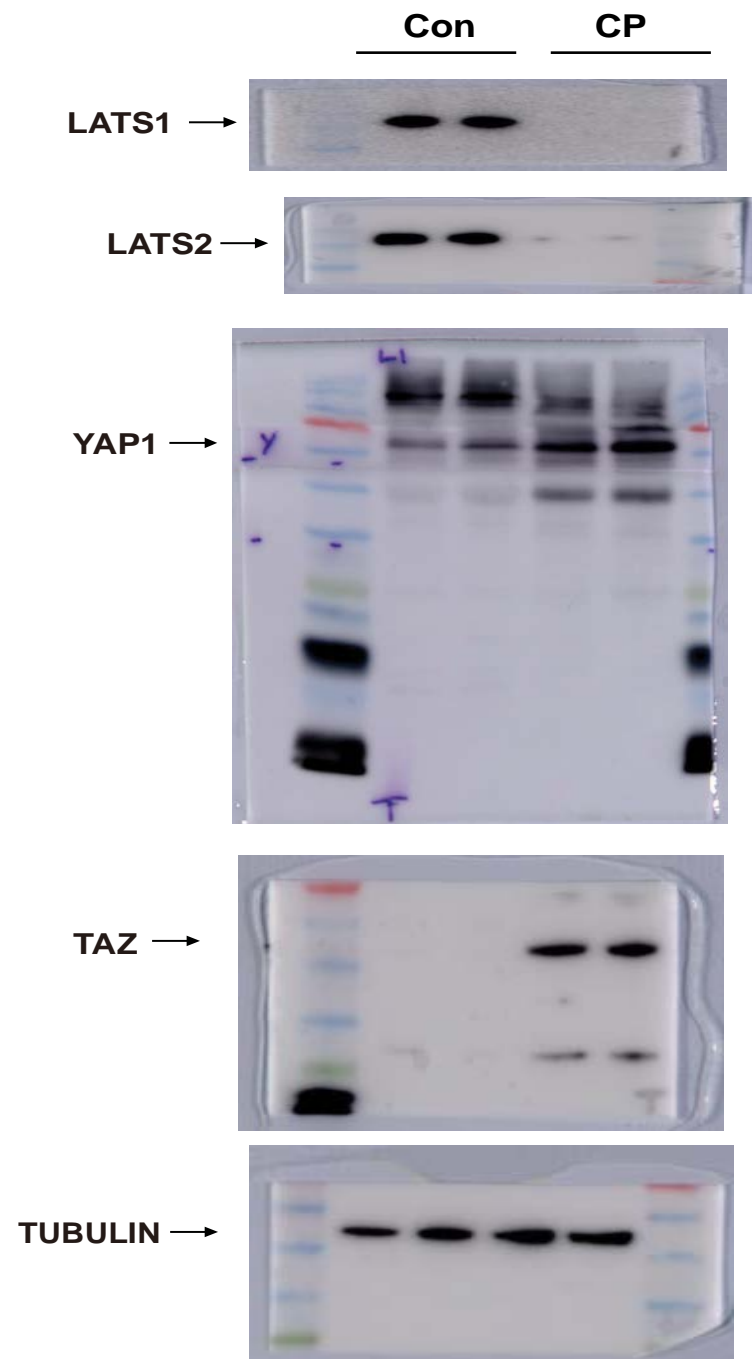

Supplement: S1 Raw Images — (PDF) [file pbio.3000418.s016.pdf]
