# Supplementary material for: Activation of the intrinsic fibroinflammatory program in adult pancreatic acinar cells triggered by Hippo signaling disruption
Source: PLoS Biol. 2019 Sep 12;17(9):e3000418. doi: 10.1371/journal.pbio.3000418 (PMC6742234; doi:10.1371/journal.pbio.3000418)
Supplement: S1 Table — (DOCX) [file pbio.3000418.s012.docx]

S1 Table. Primer sequences used for genotyping

| Lats1  (F1+R: WT-350bp; F2+R: KO-230bp) | Lats2  (F1+R: WT-300bp; F2+R: KO-250bp) |
| --- | --- |
| F1: TTGTTGCTGGTGTTGTTT CC | F1: GCGCATGCCTTTAATCCTAGC |
| F2: AGGATGTAGTGAAGGCGTGTAAC | F2: CTATCGCTAGGCTGTTCCCAC |
| R:  AGACCTCGTCGCACAGAATG | R: CTGAGCAACGACTCCAGGAAC |
|  |  |
| YAP1  (F1+R1: WT-457bp; F1+R2: KO-338bp) | TAZ  (F1+R1: WT-496bp; F1+R2: KO-704bp) |
| F1:ACATGTAGGTCTGCATGCCAGAGGAGG | F1: GGCTTGTGACAAAGAACCTGGGGCTATCTGAG |
| R1:AGGCTGAGACAGGAGGATCTCTGTGAG | R1: CCCACAGTTAAATGCTTCTCCCAAGACTGGG |
| R2:TGGTTGAGACAGCGTGCACTATGGAGC | R2: AACTGCTAACGTCTCCTGCCCCTGACCTCTC |
