# Supplementary material for: Activation of the intrinsic fibroinflammatory program in adult pancreatic acinar cells triggered by Hippo signaling disruption
Source: PLoS Biol. 2019 Sep 12;17(9):e3000418. doi: 10.1371/journal.pbio.3000418 (PMC6742234; doi:10.1371/journal.pbio.3000418)
Supplement: S2 Table — (DOCX) [file pbio.3000418.s013.docx]

S2 Table. List of antibodies used in this study

| **Primary Antibody** | **Catalog** | **Dilution** | **Company** | **Application** |
| --- | --- | --- | --- | --- |
| Lats1 (C66B5) | 3477 | 1:1000 | Cell signaling technology | WB |
| Last2 | A300-479A | 1:1000 | Bethyl Laboratories,INC. | WB |
| YAP1 | 13584-1-AP | 1:1000 | Proteintech^TM^ | WB |
| P-YAP1(Ser127) | 4911S | 1:1000 | Cell signaling technology | WB |
| TAZ | 23306-1-AP | 1:1000 | Proteintech^TM^ | WB |
| CTGF (l-20) | sc-14939 | 1:1000 | Santa Cruz Biotechnology | WB |
| γ-H2AX | 2577S | 1:1000 | Cell signaling technology | WB |
| GAPDH | sc-32233 | 1:200 | Santa Cruz Biotechnology | WB |
| Tubulin | 11224-1-AP | 1:1000 | Proteintech^TM^ | WB |
| YAP1(D8H1X) | 14074 | 1:200 | Cell signaling technology | IHC |
| TAZ | HPA007415-100UL | 1:600 | Sigma-Aldrich | IHC |
| F4/80 [CI:A3-1 clone] | Ab6640 | 1:100 | Abcam | IHC |
| SPP1 | AF808 | 1:200 | R&D | IHC, IF |
| CK19 | TROMA-III | 1:50 | DSHB | IHC, IF |
| CD45 (Clone 30-F11) | 70-0451 | 1:100 | Tonbo Biosciences | IHC, IF |
| α-SMA | 14-9760-80 | 1:3000 | eBioscience | IHC, IF |
| Anti-GFP | GFP-1020 | 1:500 | AVES LABS,INC. | IF |
| Amylase (C-20) | sc-12821 | 1:100 | Santa Cruz Biotechnology | IF |
| Collagen I | ab21286 | 1:100 | Abcam | IF |
| Ki67 | RM-9106-S | 1:100 | Thermo Scientific | IF |
| Activated Caspase 3 | G748A | 1:250 | Progema | IF |
| APC-CD45 | 103111 | 1:100 | Biolegend | FC |
| BV421-F4/80 | 123137 | 1:100 | Biolegend | FC |
| Percp/Cy5.5-CD11b | 101228 | 1:100 | Biolegend | FC |
