# Supplementary material for: Activation of the intrinsic fibroinflammatory program in adult pancreatic acinar cells triggered by Hippo signaling disruption
Source: PLoS Biol. 2019 Sep 12;17(9):e3000418. doi: 10.1371/journal.pbio.3000418 (PMC6742234; doi:10.1371/journal.pbio.3000418)
Supplement: S3 Table — (DOCX) [file pbio.3000418.s014.docx]

S3 Table. Primer sequences used for Real-time PCR in this study

| Gene name | Forward | Reverse |
| --- | --- | --- |
| Lats1 | GCGATGTCTAGCCCATTCTC | GGTTGTCCCACCAACATTTC |
| Lats2 | AGCCTGACAACATACTCATCG | AATCCAGTGCAGAGGCCAAA |
| YAP1 | TACTGATGCAGGTACTGCGG | TCAGGGATCTCAAAGGAGGAC |
| TAZ | GAAGGTGATGAATCAGCCTCTG | GTTCTGAGTCGGGTGGTTCTG |
| CTGF | GGCCTCTTCTGCGATTTCG | GCAGCTTGACCCTTCTCGG |
| SPP1 | AGCAAGAAACTCTTCCAAGCAA | GTGAGATTCGTCAGATTCATCCG |
| CXCL12 | TGCATCAGTGACGGTAAACCA | TTCTTCAGCCGTGCAACAATC |
| CXCL16 | CCTTGTCTCTTGCGTTCTTCC | TCCAAAGTACCCTGCGGTATC |
| TGF-β1 | CCACCTGCAAGACCATCGAC | CTGGCGAGCCTTAGTTTGGAC |
| TGF-β2 | CTTCGACGTGACAGACGCT | GCAGGGGCAGTGTAAACTTATT |
| TGF-β3 | GGACTTCGGCCACATCAAGAA | TAGGGGACGTGGGTCATCAC |
| PDGF | GAGGAAGCCGAGATACCCC | TGCTGTGGATCTGACTTCGAG |
| YM1 | TGGTGAAGGAAATGCGTAAA | GTCAATGATTCCTGCTCCTG |
| IL-10 | GCTGGACAACATACTGCTAACC | ATTTCCGATAAGGCTTGGCAA |
| CCL17 | TACCATGAGGTCACTTCAGATGC | GCACTCTCGGCCTACATTGG |
| TNF-α | CCAAAGGGATGAGAAGTTCC | CTCCACTTGGTGGTTTGCTA |
| SOCS1 | CTGCGGCTTCTATTGGGGAC | AAAAGGCAGTCGAAGGTCTCG |
| NOS2 | GTTCTCAGCCCAACAATACAAGA | GTGGACGGGTCGATGTCAC |
| GAPDH | AGGTCGGTGTGAACGGATTTG | GGGGTCGTTGATGGCAACA |
